# Molecular arrangements that accompany binding of rice xylanase inhibitor protein OsXIP and the *Rhizopus oryzae* GH11 xylanase RXyn2

**DOI:** 10.1016/j.jbc.2025.110385

**Published:** 2025-06-16

**Authors:** Takayuki Ohnuma, Jun Tanaka, Harutada Ozaki, Keigo Mitsui, Daichi Tsujitsugu, Miki Okugawa, Toru Takeda, Makoto Ihara, Tamo Fukamizo, Daijiro Takeshita

**Affiliations:** 1 Department of Advanced Bioscience, Kindai University, Nara, Japan; 2 Agricultural Technology and Innovation Research Institute, Nara, Japan; 3 Department of Applied Biological Chemistry, Kindai University, Nara, Japan; 4 School of Biomolecular Science and Engineering (BSE), Vidyasirimedhi Institute of Science and Technology (VISTEC), Rayong, Thailand; 5 Biomedical Research Institute, National Institute of Advanced Industrial Science and Technology (AIST), Tsukuba, Ibaraki, Japan

**Keywords:** Xylanase inhibitor protein, GH18 chitinase, pathogenesis-related protein, glycoside hydrolase, hemicellulose

## Abstract

Plants have evolved xylanase inhibitor proteins as part of their defense mechanisms against phytopathogens. The rice xylanase inhibitor protein (OsXIP) is structurally similar to GH18 chitinase and homologous to wheat XIP-type inhibitor (XIP-I), which inhibits both GH10 and GH11 xylanases. Various inhibition and interaction analyses showed that OsXIP competitively inhibits the hydrolytic activity of GH11 xylanase RXyn2, but not the activity of GH10 xylanase RXyn1 from *Rhizopus oryzae*. The crystal structure of the OsXIP/RXyn2 complex showed that OsXIP, which has a (***β***/***α***)_8_-barrel fold, extrudes the loop between ***α***4 and ***β***5 (L*α*4***β***5_OsXIP_) and inserts the loop into the xylotriose binding site (−3 to −1 subsite) formed by the inner ***β***-sheet (palm) of RXyn2 jelly roll. The guanidyl group of Arg155 in L***α***4***β***5_OsXIP_ was shown to be critical for the inhibitory activity by mutational analysis. Notably, in the complex structure, the cylindrical cavity formed by the palm of RXyn2 jelly roll stacked upright on the loops at the N terminal ends of the ***β***-strands of OsXIP (I-formation). On the other hand, in the complex structure of XIP-I and GH11 xylanase from *Talaromyces funiculosus* (XYNC), the cavity of XYNC laid tangentially to the part of the corresponding region of XIP-I through the L*α*4***β***5_XIP-I_ (T-formation). The dissociation constant of the OsXIP/RXyn2 complex was one-tenth of that of the XIP-I/XYNC complex (4.2 *versus* 41.5 nM). OsXIP may have adapted to bind and inhibit GH11 enzymes, which are resistant to the inhibition by XIP-I type proteins, by changing its binding mode.

Rice grain is a staple food for more than half of the world's population. It has been an important issue to grow rice plants steadily even on earth undergoing various environmental problems (1, 2). However, infection of rice plants by a variety of pathogens, including viruses, bacteria, fungi, and pests hampers their food quality and brings about significant yield loss annually in many rice-growing countries (3, 4). Plants possess pathogenesis-related proteins, called PR proteins, to protect themselves from invasive pathogens using these proteins. In the current classification, PR proteins are grouped into 17 families (PR-1 to PR-17) based on their molecular structure and biological function (5, 6, 7, 8). Among them, chitinases (EC 3.2.1.14), which belong to families PR-3, PR-8, and PR-11, are one of the most studied kinds of PR proteins and produced by a broad range of crop and noncrop species, although plants lack chitin, a linear polysaccharide made of *N*-acetyl-D-glucosamine residues linked by *β*-1,4 linkages, as a structural component. On the other hand, xylan is a polysaccharide backbone of hemicellulose present in the primary and secondary cell walls of plants. Therefore, xylanases secreted from phytopathogens, which hydrolyze *β*-1,4 linkages of xylan, play a major role in their infection processes. Based on amino acid sequence similarity, most fungal xylanases have been grouped into the glycoside hydrolase families 10 and 11 (GH10 and GH11) (9, 10). In fact, GH10 and GH11 enzymes secreted from *Magnaporthe oryzae* and *Botrytis cinerea* were shown to be crucial for their pathogenesis (11, 12).

Xylanase inhibitor proteins are plant protein inhibitors that suppress the hydrolytic activity of xylanases from phytopathogens. To date, three structurally different classes of inhibitors have been identified in wheat, *Triticum aestivum* xylanase inhibitor type (TAXI-type), xylanase-inhibiting protein type (XIP-type) and thaumatin-like xylanase inhibitor type (TLXI-type) (13, 14, 15). TAXI- and TLXI-types have been shown to be active only against GH11 xylanases of fungal and bacterial origins, whereas XIP-type inhibitors are able to inhibit both fungal GH10 and GH11 xylanases but inactive toward bacterial ones (15, 16, 17, 18). XIP-I is the first XIP-type inhibitor identified in wheat flour and was found to inhibit the activity of GH11 enzymes from *Aspergillus awamori* var. Awamori and *Trichoderma viride* (14). It was also found to inhibit the activity of xylanases from various phytopathogens, including GH10 xylanase from *Aspergillus nidulans* (XLNC) and GH11 xylanase from *Talaromyces funiculosus* (XYNC) (17, 18). Nowadays, it is known that this type of inhibitor is widely distributed in cereals, including rye, durum wheat, barley, maize, and rice (19, 20). Although XIP-type inhibitors share significant sequence similarity with GH18 chitinases and adopt a (*β*/*α*)_8_-barrel fold, they are thought to be devoid of chitinase activity because of lack of the catalytic DxDxE motif, which is completely conserved in the catalytic domains of GH18 chitinases (14, 21, 22, 23). Therefore, XIP-type inhibitors have been suggested to play an important role in plant defense against phytopathogens in a manner distinct from plant chitinases. Plant chitinases directly attack phytopathogens by hydrolyzing their cell wall chitin, whereas XIP-type inhibitors exclusively defend plants by inhibiting their xylanases upon infection. In addition, the inhibitors are possibly responsible for regulation of the endogenous xylanases, which hydrolyze arabinoxylan in the cell walls to remodel or reconstruct them at certain stages of cell growth and development in plants.

As in the case of plant chitinases, XIP-type inhibitors are encoded by a multigene family in cereals. In the rice genome, 20 sequences out of 34 putative GH18 genes encode GH18 proteins that partially or fully lack the catalytic DxDxE motif (24, 25, 26). Among them, at least four genes, *riceXIP*, *RIXI*, *OsXIP*, and *OsHI-XIP*, have been reported to encode XIP-type inhibitors, to date (Fig. S1) (20, 27, 28, 29). Although these isoforms are assumed to have distinct and redundant functions in their inhibition selectivity and potency, such properties of individual isoforms in a single plant species remain unknown. In this study, to understand at the molecular level the self-defense strategy of rice plants against phytopathogens using XIP-type inhibitors, we evaluated the inhibitory activity and interaction ability of OsXIP against GH10 and GH11 xylanases (RXyn1 and RXyn2) from *Rhizopus oryzae* (*R. oryzae*), a potent pathogen of rice (30), and determined the crystal structures of OsXIP and OsXIP/RXyn2 complex to reveal the inhibitory mechanism. Our findings provide new insights into the molecular mechanism of GH11 xylanase inhibition by XIP-type inhibitors.

## Results

### Inhibitory activity of OsXIP on xylanases from *R. oryzae*

**Figure 1:**
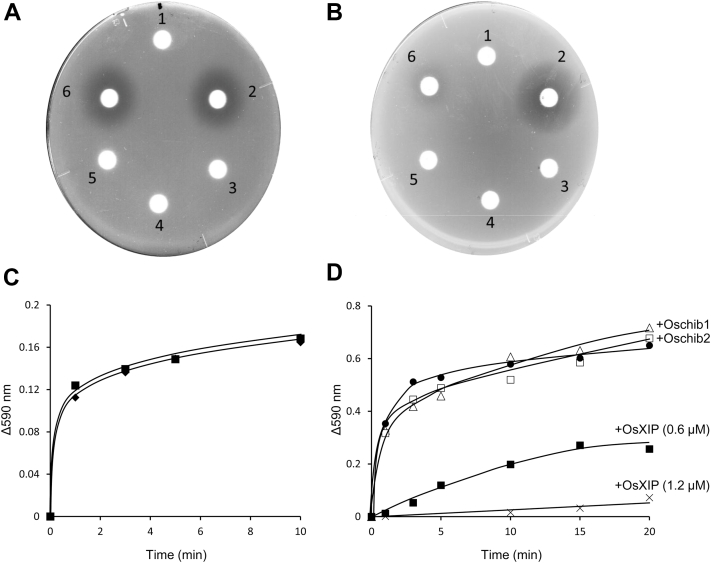
**Inhibitory activity of OsXIP on xylanases from *Rhizopus oryzae*.** Inhibition assay of OsXIP against RXyn1 and RXyn2 on 1% beechwood xylan-added agar plates. *A*, inhibitory activity of OsXIP against RXyn1. 1, control (distilled water); 2, RXyn1; 3, boiled RXyn1; 4, OsXIP; 5, mixture of OsXIP and boiled RXyn1; and 6, mixture of OsXIP and RXyn1. *B*, inhibitory activity of OsXIP against RXyn2. 1, control; 2, RXyn2; 3, boiled RXyn2; 4, OsXIP; 5, mixture of OsXIP and boiled RXyn2; and 6, mixture of OsXIP and RXyn2. Filter paper discs containing 10 μl of test proteins were placed on plates containing beechwood xylan. Petri dishes were incubated at 37 °C for 18 h. The clear halo around the disc indicates xylan degradation by the test protein. *C*, hydrolysis of RBB-xylan by RXyn1 in the absence (*closed diamonds*) and presence of OsXIP (*closed squares*). *D*, hydrolysis of RBB-xylan by RXyn2 in the absence (*closed circles*) and presence of rice GH18 proteins. Oschib1 (*open triangles*); Oschib2 (*open squares*); 0.6 μM of OsXIP (*closed squares*); and 1.2 μM of OsXIP (*crosses*). XIP, xylanase-inhibiting protein; RBB-xylan, Remazol brilliant blue-xylan.

**Figure 2:**
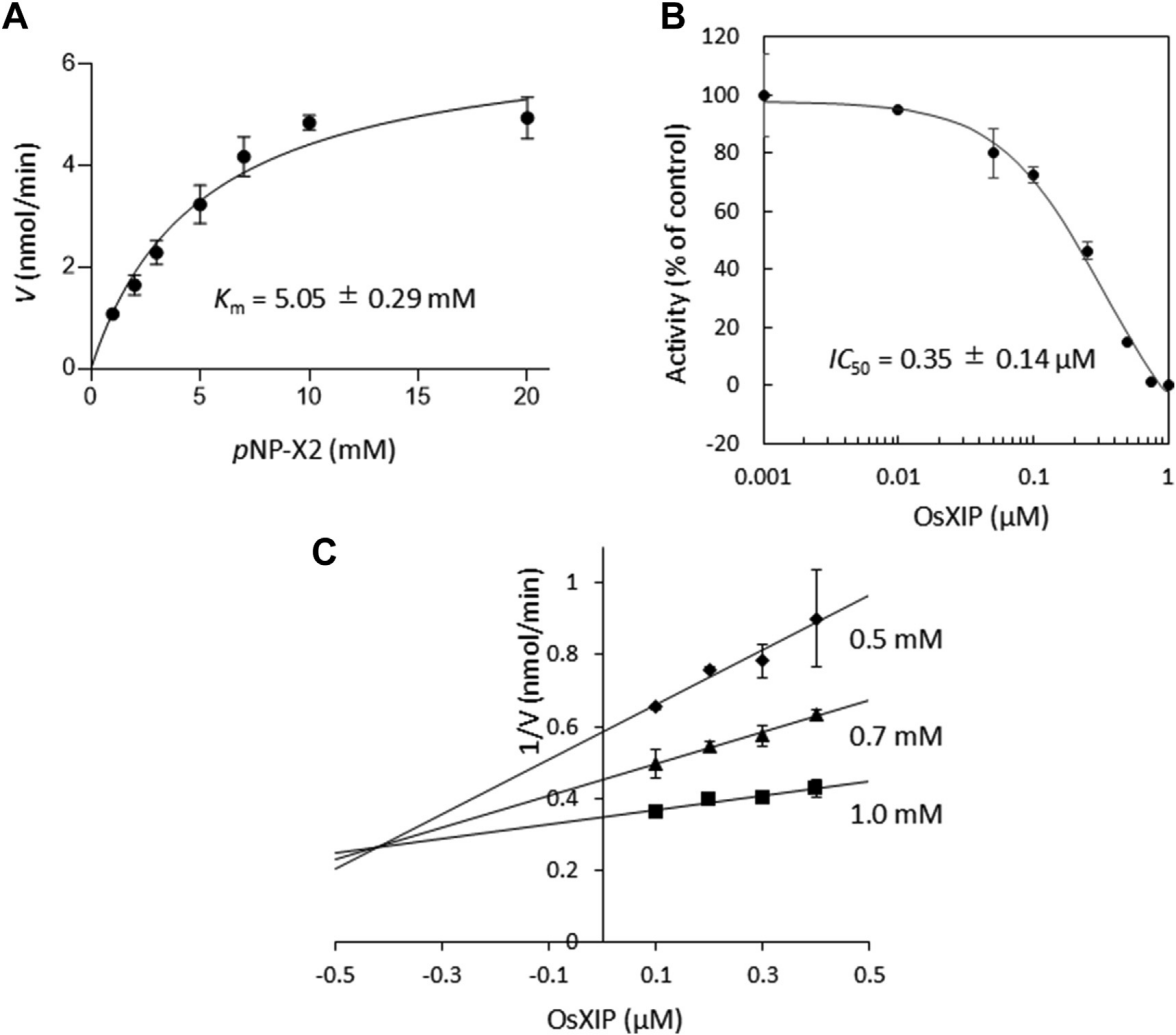
**Analysis of the inhibition of RXyn2 by OsXIP.***A*, the Michaelis–Menten plot shows the kinetic of RXyn2 on pNP-GlcNAc as substrate. *B*, dose-response curve and *IC*_50_ value of RXyn2 inhibition for OsXIP. *C*, Dixon plot was generated by determining the initial velocity against varying concentrations of OsXIP and *p*NP-X2. Error bars represent standard deviations obtained from three independent experiments.

Inhibitory activity of OsXIP on xylanases from *R. oryzae* (RXyn1 and RXyn2) was initially evaluated using an agar plate supplemented with 1% beechwood xylan. In the absence of OsXIP, clear halos were created around the discs containing RXyn1 or RXyn2, demonstrating that they both degraded beechwood xylan in the agar (discs at No. 2 in Fig. 1, *A* and *B*). A clear halo was created around the disc with a mixture of OsXIP and RXyn1 (No. 6 in Fig. 1*A*), but not around the disc with a mixture of OsXIP and RXyn2 (No. 6 in Fig. 1*B*), indicating that OsXIP inhibited the xylanase activity of RXyn2. No xylanase activity was detected in OsXIP itself (discs at No. 4 in Fig. 1, *A* and *B*). Negative controls were carried out with boiled proteins (discs at No. 3 and 5 in Fig. 1, *A* and *B*), showing no halos. Next, xylanase activity of RXyn1 and RXyn2 against the chromogenic polymer substrate Remazol brilliant blue-xylan (RBB-xylan)_ was measured in the absence and presence of OsXIP, respectively. As shown in Figure 1, *C* and *D*, OsXIP did not suppress the hydrolysis of the substrate by RXyn1 but did suppress the hydrolysis by RXyn2. The degree of suppression of the substrate hydrolysis by RXyn2 was enhanced by doubling the dose of OsXIP, suggesting that OsXIP inhibits the xylanase activity of RXyn2 in a dose-dependent manner. The inhibitory activity of two rice GH18 chitinases (Oschib1 and Oschib2) belonging to PR-8 and sharing the (*β*/*α*)_8_-barrel fold against RXyn2 was also examined for comparison (24). The GH18 chitinases did not affect the xylanase activity (Fig. 1*D*), suggesting that the inhibition of RXyn2 by OsXIP was attributed to the specificity between OsXIP and RXyn2 derived from the unique structural moiety of OsXIP, which is absent in the rice GH18 chitinases. To quantify the inhibitory activity of OsXIP on RXyn2, a kinetic analysis of RXyn2 was first conducted using the synthetic substrate *p*-nitrophenyl xylobioside (*p*NP-X2). Figure 2*A* illustrates the initial velocity profile of *p*NP release catalyzed by RXyn2 at different substrate concentrations ranging from 0.0 to 20 mM. The Michaelis constant (*K*_m_) was found to be 5.05 ± 0.29 mM. Next, the percentage of inhibition was plotted against OsXIP concentrations, as shown in Figure 2*B*. OsXIP inhibited RXyn2's xylanase activity in a dose-dependent manner with an *IC*_50_ value of 0.35 ± 0.14 μM. To verify the mechanism by which RXyn2 is inhibited by OsXIP, we tested the ability of various OsXIP concentrations to inhibit *p*NP-X2 hydrolysis at several fixed substrate concentrations. As shown in Figure 2*C*, plotting the inverse of the hydrolytic velocity against the inhibitor concentration at different *p*NP-X2 concentrations yielded a series of lines intersecting in the top left quadrant, suggesting a competitive inhibition mechanism. With the *IC*_50_ and *K*_m_ values for RXyn2, *K*_i_ value for competitive inhibition was calculated to be 0.29 ± 0.12 μM.

### Analysis of OsXIP binding to RXyn1 and RXyn2 using gel filtration chromatography, SDS-PAGE, and analytical ultracentrifugation

**Figure 3:**
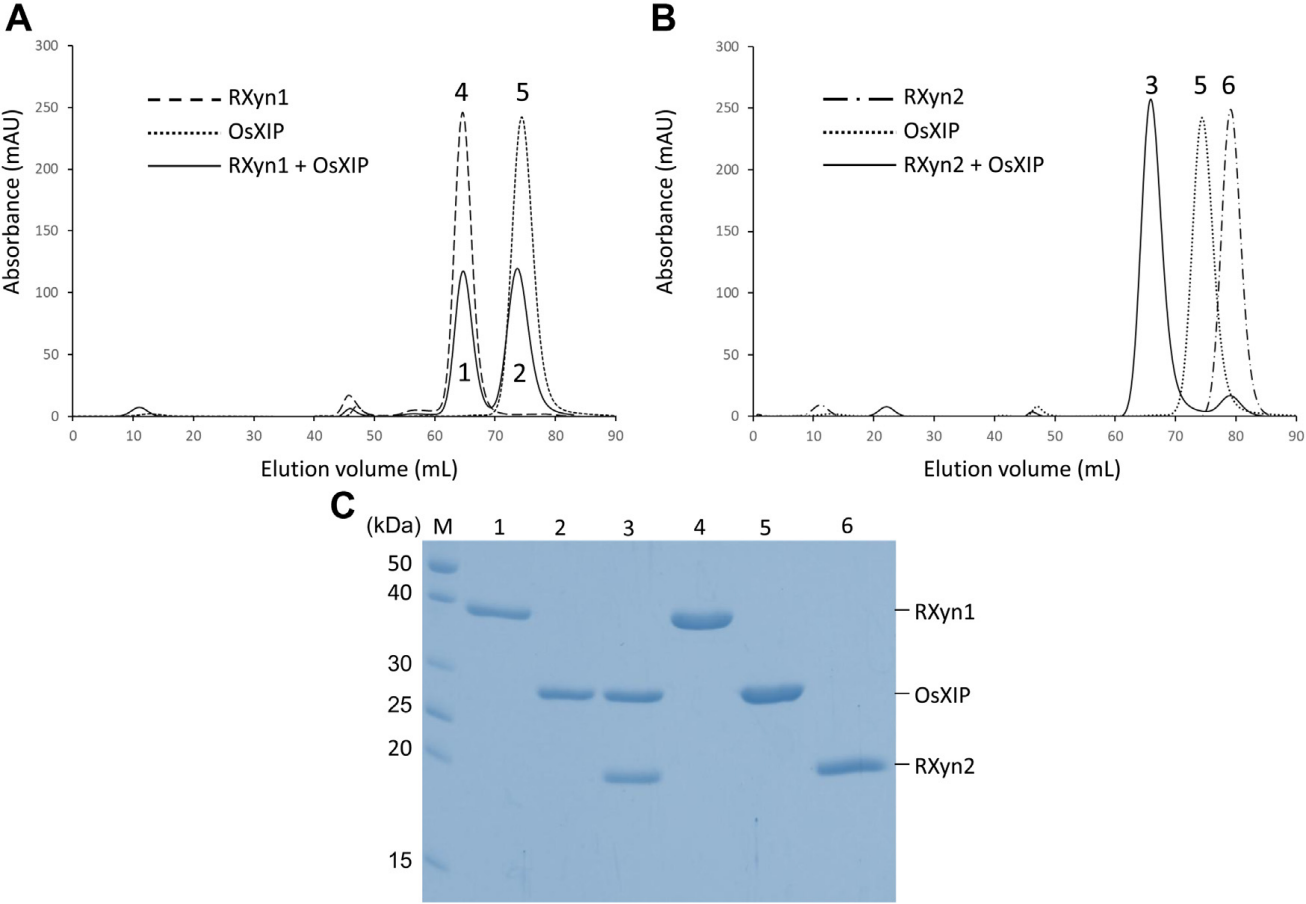
**Gel filtration analysis of the interaction of OsXIP with RXyn1 and RXyn2.***A*, chromatograms of OsXIP (*dotted line*), RXyn1 (*long-dashed line*), and mixture of OsXIP and RXyn1 (*solid line*). *B*, chromatograms of OsXIP (*dot line*), RXyn2 (*chain line*), and mixture of OsXIP and RXyn2 (*solid line*). *C*, SDS-PAGE analysis of each elution peak in (*A* and *B*). XIP, xylanase-inhibiting protein.

When OsXIP and RXyn1 were individually subjected to a gel filtration column of HiLoad 16/600 Superdex 75 pg, these proteins exhibited symmetrical peaks at retention volumes of 65 and 75 ml (peaks 4 and 5 in Fig. 3*A*), respectively, suggesting that both proteins exist as monomers in solution. When an equimolar mixture of OsXIP and RXyn1 was applied to the same column, the retention volumes were the same as when each was applied individually (peaks 1 and 2 in Fig. 3*A*). SDS-PAGE of the peak fractions (peaks 1, 2, 4, and 5) exhibited single protein bands for individual OsXIP and RXyn1 (Fig. 3*C*). On the other hand, although RXyn2 itself was eluted at a retention volume of 79 ml (peak 6 in Fig. 3*B*), an equimolar mixture of OsXIP and RXyn2 exhibited a major peak at 67 ml (peak 3 in Fig. 3*B*) together with a small peak at 79 ml. SDS-PAGE of the peak fractions (peaks 3, 5, and 6) showed that the major peak at 67 ml (peak 3) contained two protein bands, corresponding to OsXIP and RXyn2 (lane 3 in Fig. 3*C*), whereas peaks 5 and 6 exhibited single protein bands corresponding to OsXIP and RXyn2, respectively. These results indicate that OsXIP interacts with RXyn2, but not with RXyn1, in 1:1 stoichiometry to form the OsXIP/RXyn2 complex. The OsXIP/RXyn2 complex appeared to be stable during the chromatographic separation, suggesting that OsXIP binds to RXyn2 with high affinity.

OsXIP binding to RXyn1 or RXyn2 was also analyzed by sedimentation velocity experiments using an analytical ultracentrifuge. Figure S2 shows the sedimentation coefficient distributions for OsXIP, RXyn1, RXyn2, OsXIP+RXyn1, and OsXIP+RXyn2, respectively. OsXIP, RXyn1, and RXyn2 exhibited symmetrical distributions at the sedimentation coefficients of 3.11, 3.05, and 2.34 S, respectively, which are almost correlated with the calculated molecular masses for the individual proteins (31,284.28 Da, 34,011.89 Da, and 22,340.17 Da), suggesting that the proteins were sedimented in their monomeric forms (Fig. S2, *A*, *B*, and *D*). When the mixture of OsXIP and RXyn1 was applied to the sedimentation analysis, both proteins exhibited similar distributions at similar sedimentation coefficients (S = 3.06), indicating no interaction between these proteins (Fig. S2*C*). However, for the mixture of OsXIP and RXyn2, the distribution profile clearly shifted to a sedimentation coefficient of 3.89, indicating that OsXIP formed the complex with RXyn2 resulting in the higher sedimentation coefficient (Fig. S2*E*).

### Analysis of OsXIP binding to RXyn1 and RXyn2 using isothermal titration calorimetry

**Figure 4:**
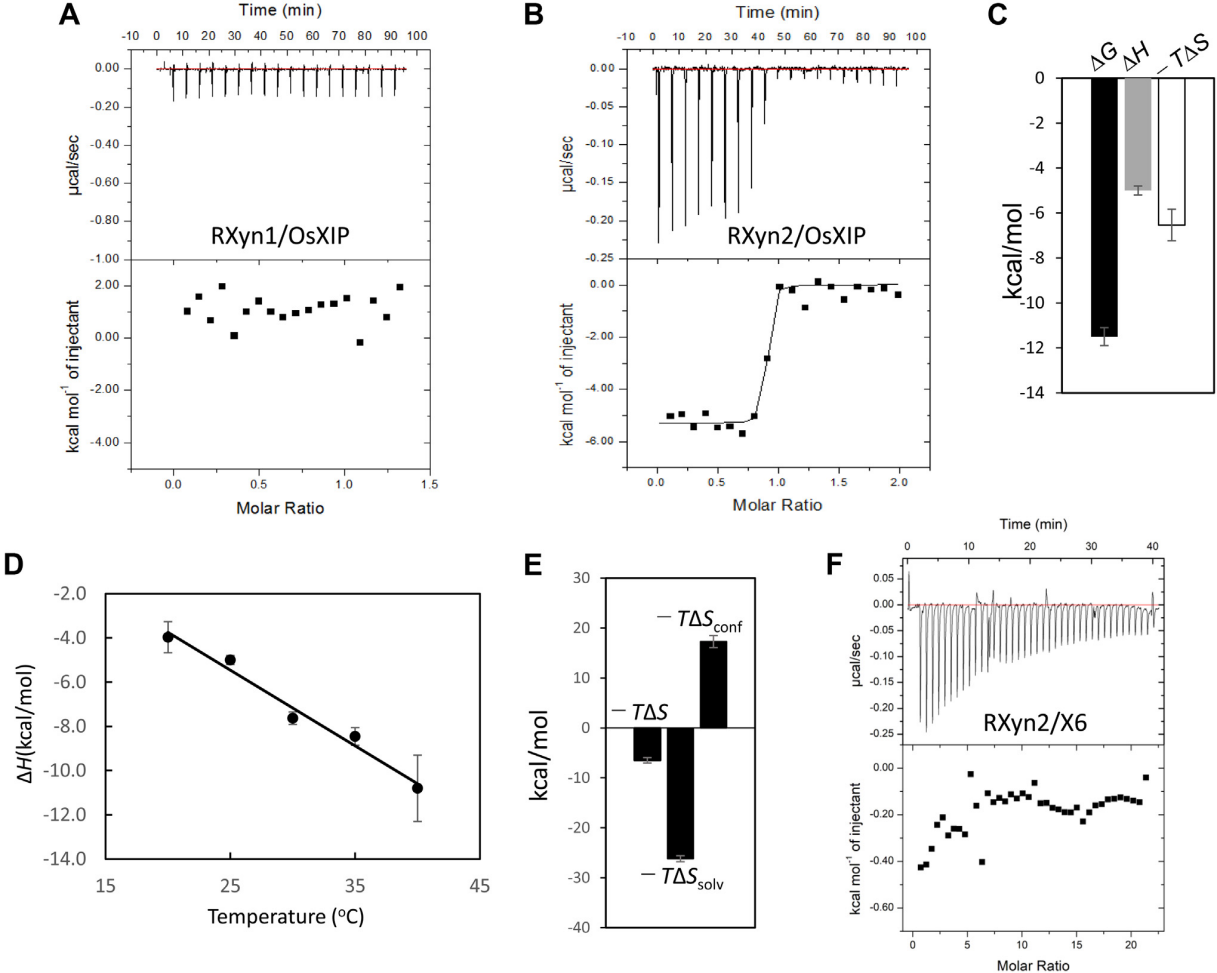
**ITC of OsXIP with RXyn1, RXyn2, and xylohexaose.***A*, ITC profile obtained with titration of OsXIP to RXyn1. *B*, ITC profile obtained for titration with OsXIP to RXyn2. Raw data (*upper panel*) and integrated heat measurements (*lower panel*) from ITC are shown. *C*, the bar diagram of thermodynamic parameters for binding of OsXIP to RXyn2 with error bars representing the standard deviation obtained from three independent experiments. *D*, temperature dependence of the enthalpy change for OsXIP binding to RXyn2. The plots of Δ*H*° *versus* temperature yielded the change in heat capacity (Δ*C*_p_°) based on the slope of the line. The Δ*C*_p_° value was calculated to be −343.3 ± 8.0 cal/K·mol. *E*, the bar diagram of entropic contributions to the binding of OsXIP to RXyn2 with error bars representing the standard deviation. −*T*Δ*S*, total binding entropy change; −*T*Δ*S*_conf_; conformational entropy change; −*T*Δ*S*_solv_, solvation entropy change. *F*, ITC profile obtained for titration of xylohexaose (X6) to RXyn2_E184Q. ITC, isothermal titration calorimetry; XIP, xylanase-inhibiting protein.

Binding of OsXIP to RXyn1 and RXyn2 was studied by isothermal titration calorimetry (ITC), which would give both binding constant and enthalpy change value when they interact with each other. Heat release or absorption was not observed by injection of OsXIP solution into RXyn1 solution throughout the experiment, indicating that they did not interact (Fig. 4*A*). In contrast, injection of OsXIP solution into RXyn2 solution produced an exothermic heat of the interaction, which gradually decreased with successive injections (Fig. 4*B*), indicating that OsXIP binds to RXyn2. The theoretical binding isotherm was satisfactorily fitted to the experimental data points by varying the binding affinity constant (*K*_a_), number of binding sites (*i.e.*, stoichiometry of the reaction (*n*)), and enthalpy change of OsXIP binding (Δ*H*°) to RXyn2 using a nonlinear least-squares algorithm. The optimized *n* value was 0.81 ± 0.058, indicating a binding stoichiometry of 1:1. At this temperature and pH (25 °C and pH 5.0), OsXIP was found to bind to RXyn2 with a *K*_d_ (=1/*K*_a_) of 4.2 ± 3.3 nM. Binding interaction was both enthalpy- and entropy-driven (Δ*H*° = −5.0 ± 0.20 kcal/mol and −*T*Δ*S*° = − 6.5 ± 0.55 kcal/mol), resulting in Δ*G*° of – 11.5 ± 0.36 kcal/mol (Fig. 4*C* and Table 1). Xylohexaose (X6) is a good substrate for GH11 xylanase and when bound occupies the entire substrate binding cleft (31). Therefore, X6 binding to RXyn2_E184Q, which is an inactive mutant of RXyn2, was also investigated by ITC. As shown in Figure 4*F*, X6 did bind to the mutant and an exothermic reaction was observed. However, the *K*_d_ for binding could not be successfully determined from this experiment because the binding interaction was weak, and the Wiseman *c*-value was less than 10. For an accurate *K*_d_ determination by ITC, the *c*-value (the product of the xylanase concentration and the binding constant, *K*_a_) must be between 10 and 100 (32).

**Table 1:** Thermodynamic parameters for the interaction of OsXIP with RXyn2

Temperature	Stoichiometry	*K*_d_ (nM)	Δ*H*^o^ (kcal/mol)	−*T*Δ*S*^o^ (kcal/mol)	Δ*G*^o^ (kcal/mol)
25	0.81 ± 0.058	4.2 ± 3.3	−5.0 ± 0.20	−6.5 ± 0.55	−11.5 ± 0.36

Heat capacity change (Δ*C*_p_°) upon protein-protein interaction is of great interest because it can give an insight into the mechanism of complex formation. The Δ*C*_p_° value for binding interaction between OsXIP and RXyn2 was calculated to be −343.4 ± 8.0 cal/K·mol from the measurements of binding enthalpy change over a temperature range from 20 to 40 °C (Fig. 4*D*). The obtained Δ*C*_p_° value allowed for parameterization of the binding entropy change and indicated that the major contributor to a favorable entropy change (−*T*Δ*S*° = −6.5 ± 0.55 kcal/mol) is the solvation entropy change (−*T*Δ*S*_solv_° = −26.2 ± 0.61 kcal/mol), which is partially compensated by an unfavorable conformational entropy change (−*T*Δ*S*_conf_° = 17.3 ± 1.2 kcal/mol) (Fig. 4*E* and Table 2). These results indicate that these two proteins bind rigidly, which leads to loss of conformational degrees of freedom, and they undergo desolvation of their binding interfaces upon the complexation.

**Table 2:** Heat capacity change (Δ*C*_p_°) for binding interaction between OsXIP and RXyn2 and dissection of the entropic term

Δ*C*_p_^o^ (cal/K·mol)	−*T*Δ*S*^o^ (kcal/mol)	−*T*Δ*S*_mix_^o^ (kcal/mol)	−*T*Δ*S*_conf_^o^ (kcal/mol)	−*T*Δ*S*_solv_^o^ (kcal/mol)
−343.4 ± 8.0	−6.5 ± 0.55	2.4	17.3 ± 1.2	−26.2 ± 0.61

### Crystal structure of OsXIP and comparison with XIP-I and hevamine

**Figure 5:**
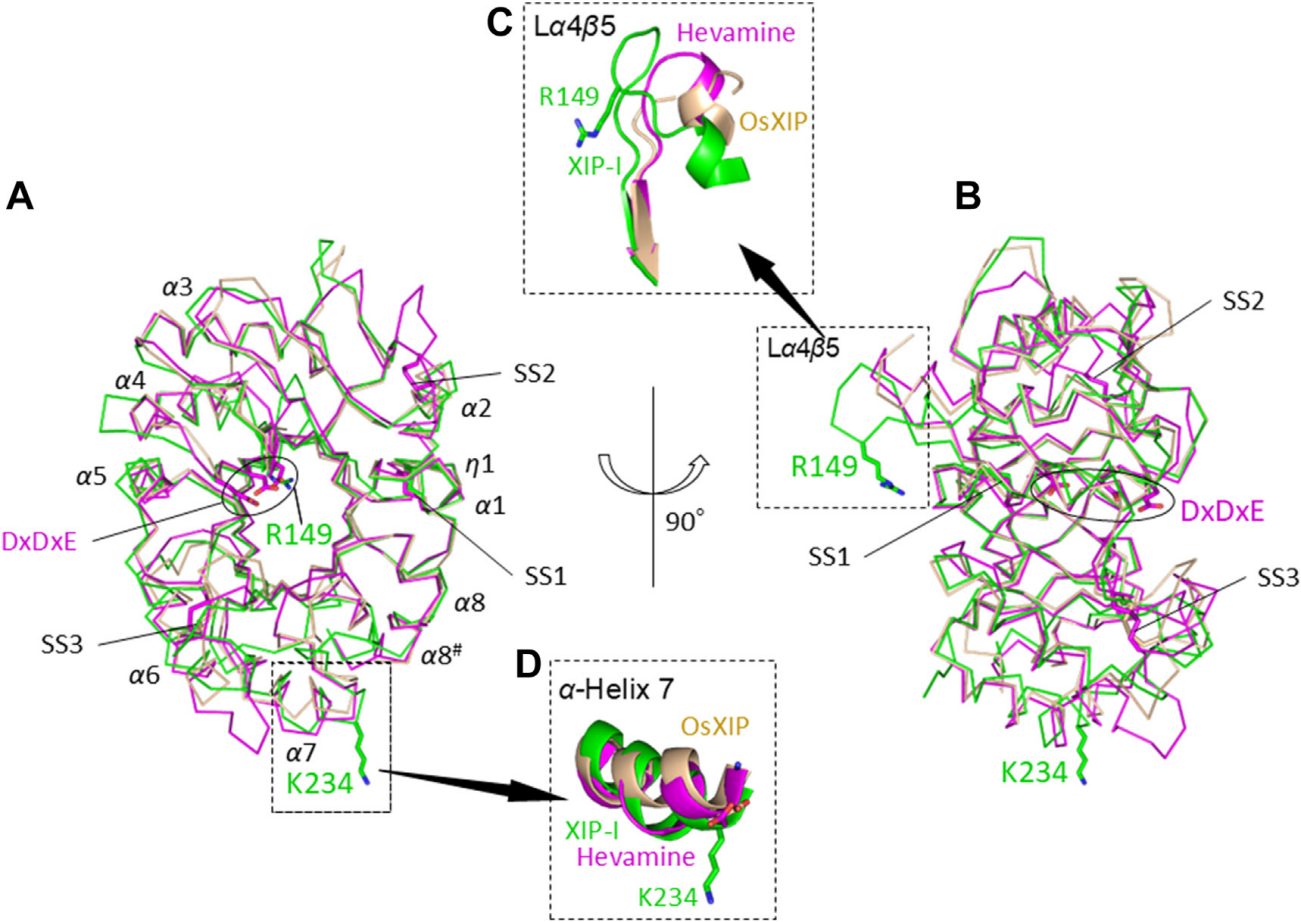
**Crystal structures of OsXIP, XIP-I, and hevamine.** Superimposed structure of OsXIP (*brown line*, PDB ID 9K1M) with XIP-I (*light green line*, PDB ID 1om0) and hevamine (*magenta line*, PDB ID 2hvm). The catalytic signature motif of GH18 chitinases, DxDxE, in hevamine is shown using *sticks*. *A*, *top* view of the superimposed structure. *α*-Helix and disulfide bond are indicated by *α* and SS, respectively. *B*, the side view of the superimposed structure, rotated by 90° about the vertical axis relative to *A*. The *right* side is the *top* of the barrel, while the *left* is the *bottom*. *C*, superimposed structure of L*α*4*β*5 region of OsXIP with those of XIP-I and hevamine shown in cartoon representation. Arg149_XIP-I_ is shown using sticks. *D*, superimposed structure of *α*-helix seven of OsXIP with those of XIP-I and hevamine shown in cartoon representation. Lys234_XIP-I_, Glu237_OsXIP_, and Asp233_hev_ are shown using *sticks*. PDB, Protein Data Bank; XIP, xylanase-inhibiting protein.

Crystal structure of OsXIP was determined using the molecular replacement method, with the structure of XIP-I from wheat (Protein Data Bank (PDB) ID 1om0) as a search model (23). The OsXIP structure was refined to *R*_work_/*R*_free_ of 16.6%/17.9% at a resolution of 1.30 Å (Table 3) and shown to adopt a (*β*/*α*)_8_-barrel fold, as observed in family GH18 chitinase structures (21, 22). Electron density maps of the amino acid residues (Arg155-Val158) consisting of a part of the loop between *α*4 and *β*5 (L*α*4*β*5_OsXIP_) were unobservable in the structure. Although OsXIP shares only 38.2% and 27.6% sequence identity, respectively, with XIP-I and hevamine, a GH18 chitinase from rubber tree *Hevea brasiliensis*, the main chain structure of OsXIP was closely similar to those of XIP-I and hevamine with RMSDs of 0.85 and 0.77 Å for the corresponding 260 and 253 Cα atoms, respectively (20, 22) (Fig. 5). The structure of hevamine has a shallow substrate binding groove on top of the barrel structure, where several amino acid residues, Gln9, Asn34, Asn45, Gly81, Ile82, Asp125, Glu127, Tyr183, Ala224, and Trp255, form hydrogen bonds with allosamidin, a pseudo-trisaccharide potent GH18 chitinase inhibitor, when complexed (Fig. S3) (21). However, in the OsXIP structure, these amino acid residues were substituted, that is, Gln9→Arg18, Asn34→Ser43, Asn45→Asp55, Gly81→Gln85, Ile82→Gly86, Asp125→Phe131, Glu127→Asp133, Tyr183→Phe192, Ala224→Val228, and Trp255→Trp258, respectively, and the substitutions appeared to have caused the protein to lose binding ability of allosamidin. The catalytic signature motif, DxDxE, conserved in GH18 chitinases, was found in center of the groove of hevamine, but not in the structure of OsXIP. In most GH18 chitinases, three disulfide bonds (SS1, SS2, and SS3) are conserved; however, only the two disulfide bonds corresponding to SS1 and SS3 of hevamine are formed in the structure of OsXIP. SS1 (Cys29-Cys71) connects the helices *α*1 and *α*2, while SS3 (Cys168-Cys197) connects the loop between *β*5 and *α*5 and the loop between *β*6 and *α*6 in OsXIP (Fig. 5, *A* and *B*).

**Table 3:** Data collection and refinement statistics

	OsXIP	OsXIP/RXyn2 complex
Data collection		
Wavelength (Å)	0.9800	0.9800
Space group	*P*2_1_2_1_2_1_	*I*422
Unit-cell parameters		
*a*, *b*, *c* (Å)	*a* = 61.35, *b* = 68.74, *c* = 81.13	*a* = 197.56, *b* = 197.56, *c* = 136.32
*α*, *β*, *γ* (º)	*α* = 90, *β* = 90, *γ* = 90	*α* = 90, *β* = 90, *γ* = 90
Resolution (Å)	50.0–1.30	20.0–2.20
	(1.37–1.30)	(2.33–2.20)
Completeness (%)	99.1 (95.1)	99.8 (99.9)
*R*_sym_	0.053 (0.863)	0.111 (1.073)
*I*/σ (*I*)	15.46 (1.42)	15.35 (2.29)
Redundancy	6.6 (5.5)	10.4 (10.6)
*CC*_1/2_	0.999 (0.696)	0.999 (0.853)
Refinement		
Resolution (Å)	40.6–1.30	19.9–2.20
No. of reflections	161,647	67,529
*R*_work_/*R*_free_ (%)	16.6/17.9	26.7/31.7
No. of atoms		
Protein	2069	7178
Water	244	276
*B*-factors (Å^2^)		
Protein	20.7	50.3
Water	35.4	45.9
R.m.s.d’s		
Bond lengths (Å)	0.014	0.002
Bond angles (°)	1.34	0.55
Ramachandran plot		
Favored region (%)	98.1	95.0
Allowed region (%)	1.9	5.0
Disallowed region (%)	0	0

Values in parentheses are for the highest resolution shell.

### Crystal structure of OsXIP/RXyn2 complex

**Figure 6:**
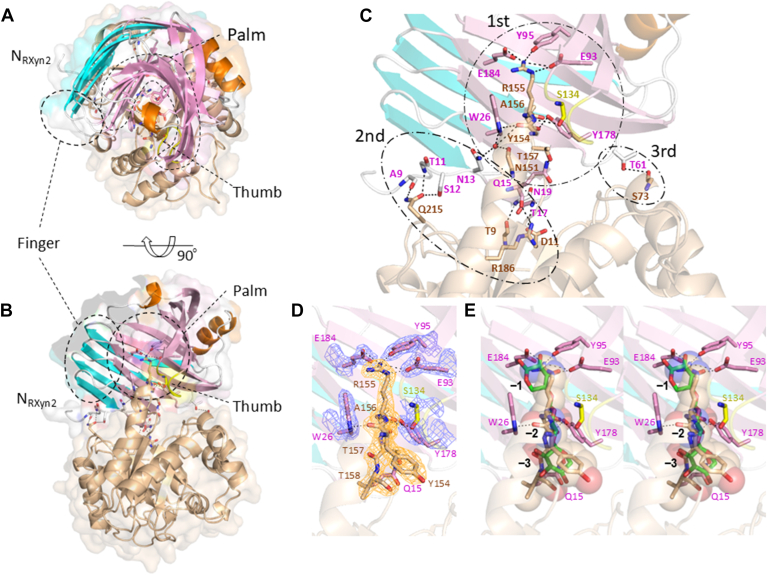
**Crystal structure of OsXIP in complex with RXyn2.** Cartoon and transparent surface representations of the OsXIP/RXyn2 complex (PDB ID 9K1N). OsXIP is colored *brown*. The outer and inner *β*-sheets and thumb of RXyn2 are colored *cyan*, *pink*, and *yellow*, respectively. The palm and finger regions are surrounded by *dotted lines* and labeled. Helices of RXyn2 are colored *orange*. Amino acid residues involved in the interaction between OsXIP and RXyn2 are indicated using *sticks* and labeled. Water molecule was shown by *red sphere*. Hydrogen bonds are represented as *dotted lines*. N_Rxyn2_ represents the N terminus of RXyn2. *A*, *top* view of the OsXIP/RXyn2 complex structure. *B*, side view of the complex structure. *C*, detailed view of the interface between OsXIP and RXyn2. The primary, secondary, and tertiary contact areas are labeled 1st, 2nd, and 3rd, respectively, and are surrounded by chain lines. *D*, 2*F*_o_-*F*_c_ map contoured at 2.0 sigma showing the electron density for a part of L*α*4*β*5 and selected residues in the substrate binding cleft of RXyn2. *E*, stereo view of the interface with xylotriose (shown in *green sticks*) superimposed from the structure of XynII liganded with xylotriose (PDB ID 6JWB). A part of L*α*4*β*5 is depicted in transparent *spheres* and *sticks*. The numbers −3 to −1 indicate the subsite positions. PDB, Protein Data Bank.

Crystal structure of OsXIP in complex with RXyn2 was solved at 2.2 Å. The relatively high *R*_work_/*R*_free_ values of the final model of the OsXIP RXyn2 complex may be due to the quality of the X-ray diffraction data with relatively high *R*_sym_ (Table 1). OsXIP was found to form a 1:1 stoichiometric complex with RXyn2, showing a small, compact structure with a conserved jelly roll fold in GH11 enzymes. The structure was likened to a partially folded human right-hand with a palm, thumb, and finger regions and has the substrate binding cleft that runs across an entire face of the inner *β*-sheet (palm) as seen in the other GH11 enzymes (Fig. 6, *A* and *B*). In the OsXIP/RXyn2 complex, the cylindrical cavity formed by the palm of RXyn2 was upright and stacked on the loops at the N terminal ends of the *β*-strands of OsXIP. There were three contact regions for the interactions between OsXIP and RXyn2 (Fig. 6*C*). The primary contact region included the interface between L*α*4*β*5_OsXIP_ extruded from the (*β*/*α*)_8_-barrel of OsXIP and the presumed glycon binding site (subsites −3, −2, and −1) of RXyn2 formed by the palm. Residues 154 to 158 in L*α*4*β*5_OsXIP_ intruded into the cleft from the edge and reached to the catalytic center of RXyn2. They formed salt bridges and hydrogen bonds with several amino acid residues in the cleft. Family GH11 xylanases are retaining enzymes and are known to follow a double-displacement mechanism, with one Glu residue acting as a general acid/base catalyst and the other as a nucleophile (31). By analogy with the other GH11 xylanases, Glu93_RXyn2_ and Glu184_RXyn2_, which are in the center of the palm (Fig. 6*C*), correspond to the residues that function as a nucleophile and a general acid/base catalyst, respectively. In detail, the carbonyl oxygen of Tyr154_OsXIP_ hydrogen bonded to N^ε1^ of Trp26_RXyn2_. The N^η2^ of Arg155_OsXIP_ made salt bridges with Glu93_RXyn2_ O^ε2^ and Glu184_RXyn2_ O^ε2^ and hydrogen bonded to the phenolic oxygen of Tyr95_RXyn2_, which are highly conserved among GH11 enzymes, respectively. The N^ε^ of Arg155_OsXIP_ formed a hydrogen bond with Glu93_RXyn2_ O^ε2^ and the main-chain N hydrogen bonded to the main chain O of Ser134_RXyn2_ in the thumb of RXyn2. The aliphatic moieties of Arg155_OsXIP_ and Ala156_OsXIP_ stacked on the indole ring of Trp26_RXyn2_. A water molecule was found to be hydrogen bonded with the carbonyl oxygens of Asn151_OsXIP_ and Tyr154_OsXIP_, the N^ε2^of Asn13_RXyn2_ and the N^ε1^ of Trp26_RXyn2_. The carbonyl oxygen of Ala156_OsXIP_ and Thr157_OsXIP_ O^γ1^ hydrogen bonded to Tyr178_RXyn2_ O^η^ and Gln15_RXyn2_ N^ε2^, respectively.

In the secondary contact region, Thr9_OsXIP_ in the N terminal loop, Asp11_OsXIP_ at the N terminal end of *β*1_OsXIP_, Arg186_OsXIP_ at the N terminal end of *β*6_OsXIP_, and Gln215_OsXIP_ in L*α*6*β*7_OsXIP_, which were all located at the N terminal side of the *β*-strands, contributed to the interaction with RXyn2. Specifically, the main-chain O of Thr9_OsXIP_, Asp11_OsXIP_ O^δ2^, Arg186_OsXIP_ N^η2^, and Gln215_OsXIP_ N^ε2^ and O^ε1^ formed hydrogen bonds with the amide nitrogens of Asn19_RXyn2_ and Thr17_RXyn2_, main-chain O of Gln15_RXyn2_, main-chain O of Ala9_RXyn2_, Ser12_RXyn2_ O^γ^ and main-chain N of Thr11_RXyn2_ located at the side of the pinky finger of RXyn2, respectively. In the tertiary contact region, only one hydrogen bond between the carbonyl oxygen of Ser73_OsXIP_ and Thr61_RXyn2_ O^γ^ was formed.

**Figure 8:**
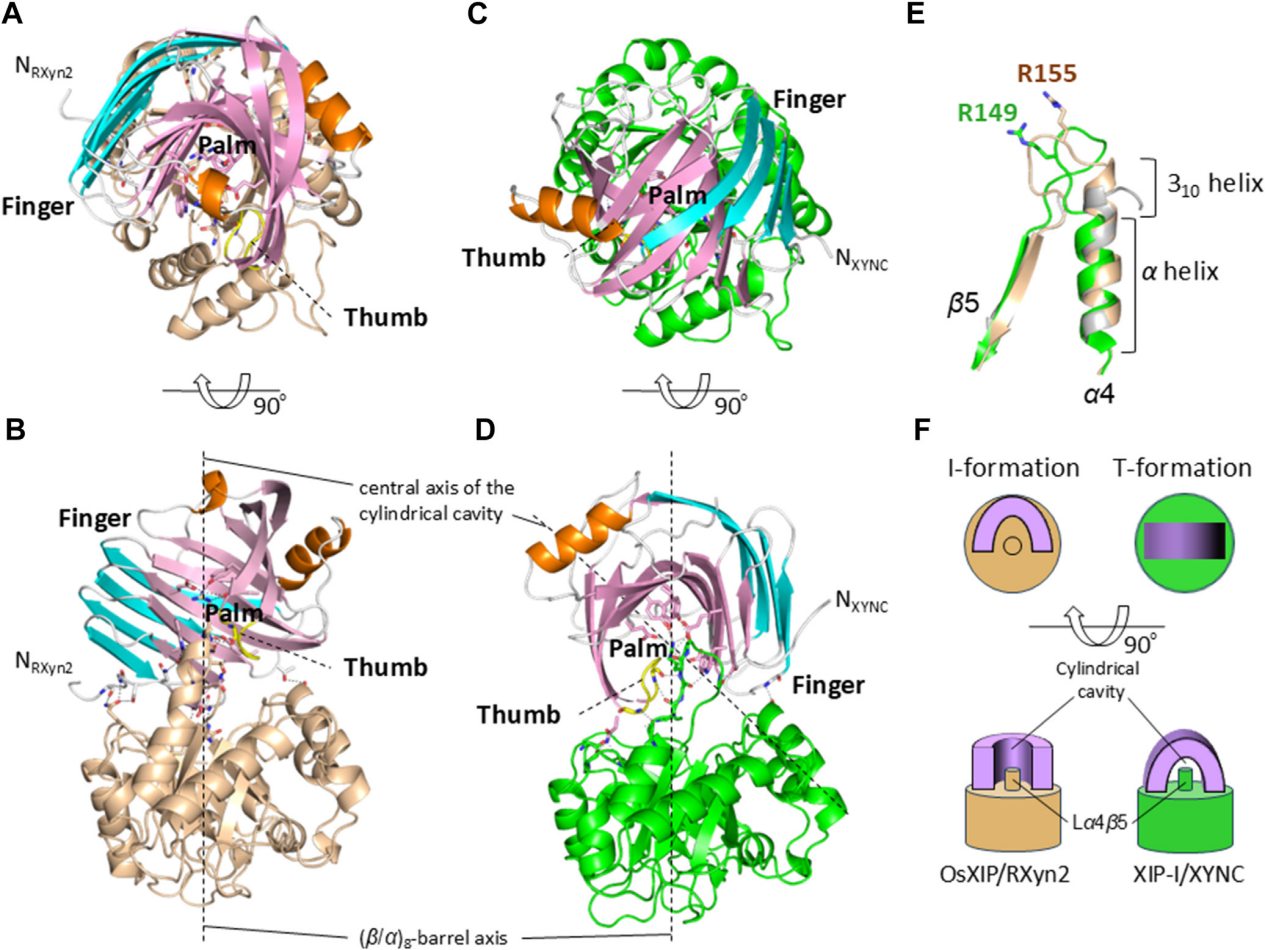
**Comparison of the crystal structure of the OsXIP/RXyn2 complex with that of the XIP-I/XYNC complex.** The complex structures are shown using cartoon representations. *A* and *B*, top and side views of the OsXIP/RXyn2 complex structure. *C* and *D*, top and side views of the XIP-I/XYNC complex structure. OsXIP and XIP-I are colored *brown* and *light green*, respectively. The outer and inner *β*-sheets and thumbs of RXyn2 and XYNC are colored as in Figure 6 and labeled. Amino acid residues directly involved in the interactions between the two proteins are depicted using *sticks*, respectively. N_RXyn2_, N terminus of RXyn2; N_XYNC_, N terminus of XYNC. The barrel axis of the XIPs and the central axis of the GH11s′ cylindrical cavity are depicted by *dotted lines*. *E*, structural alignment of L*α*4*β*5_OsXIP_ in the free OsXIP (*gray*), L*α*4*β*5_OsXIP_ in the OsXIP/RXyn2 complex (*brown*), and L*α*4*β*5_XIP-I_ in the XIP-I/XYNC complex (*light green*). Arg155_OsXIP_ and Arg149_XIP-I_, which are key residues for the interactions with RXyn2 and XYNC, are shown using *sticks*. *F*, schematic representations of top and side views of the OsXIP/RXyn2 complex in I-formation and the XIP-I/XYNC complex in T-formation. XIP, xylanase-inhibiting protein.

The structure of RXyn2 in complex with OsXIP was well superimposed with the structure of free GH11 xylanase from *Talaromyces cellulolyticus* (PDB ID 3WP3), the structure of XynII, a GH11 secreted by *Trichoderma reesei*, in complex with xylotriose (PDB ID 6JWB), and the structure of XYNC, a GH11 from *T. funiculosus*, complexed with XIP-I (PDB ID 1TE1) with the RMSD values of 0.66 Å, 0.63 Å, and 0.64 Å for the comparable Cα atoms, respectively (33, 34, 35). These features suggested that RXyn2 does not undergo substantial conformational changes upon complexation with the substrate and the inhibitor protein. The structure of free OsXIP was also well superimposed on that of OsXIP in complex with RXyn2, with a RMSD value of 0.55 Å for the comparable Cα atoms, suggesting that no major conformational changes take place following the complex formation with RXyn2. Of note, the electron density of a part of L*α*4*β*5_OsXIP_ (Arg155-Val158) was not observed in the free OsXIP structure but were clearly observed in the complex structure (Fig. 6*D*). Furthermore, the 3_10_ helix (*η*2_OsXIP_) following the *α*4 was slightly extended in the C terminal direction (Fig. 8*E*).

Upon complexation, the buried solvent accessible surface area was calculated to be 2213.9 Å^2^. The interface area between OsXIP and RXyn2 was 1107.0 Å^2^, where the polar and nonpolar buried surface areas were 562.3 Å^2^ and 544.6 Å^2^, respectively, indicating more than half of the buried surface arises from polar and charged atoms. When superimposed with xylotriose complexed with XynII (PDB ID 6JWB), L*α*4*β*5_OsXIP_ clearly occupied the glycon binding site (subsites from −3 to −1) of RXyn2. The side chain N^ε^, the carbonyl carbon of Arg155_OsXIP_, C^β^ and the carbonyl oxygen of Ala156_RXyn2_ were located close to the position of C-3 of xylose residue bound to the subsite −1, the positions of pyranose ring oxygen, O-2 and O-3 of xylose residue bound to the subsite −2, respectively (Fig. 6*E*). These results indicated that the binding mode of L*α*4*β*5_OsXIP_ partially mimics the binding mode of xylotriose to the −3 to −1 sites as observed in the complex structure of XynII-xyloriose (35).

### Mutational analysis of OsXIP

**Figure 7:**
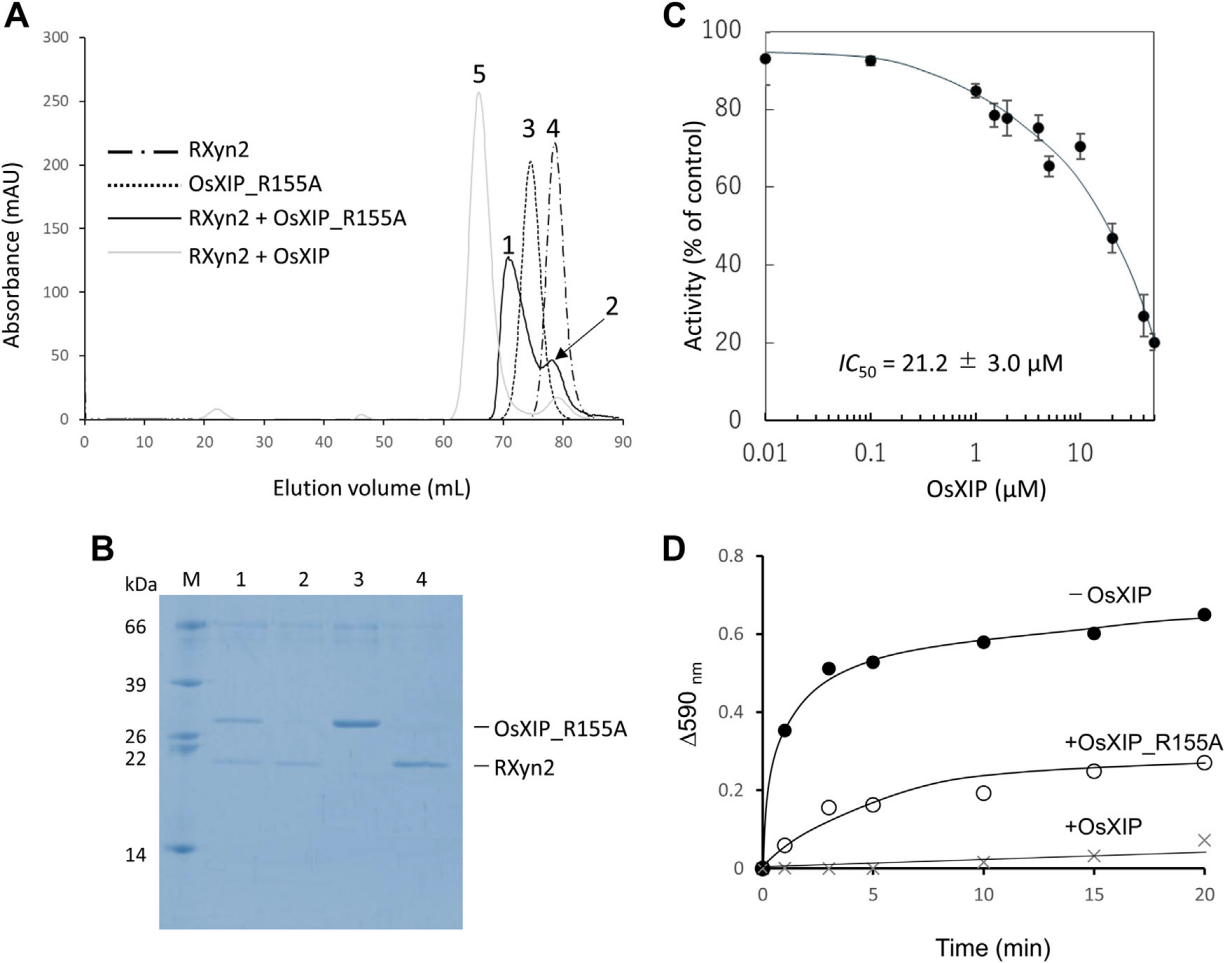
**Mutational analysis of OsXIP.***A*, gel filtration chromatography of OsXIP_R155A (*dotted line*), RXyn2 (*chain line*), mixture of OsXIP_R155A and RXyn2 (*solid line*), and mixture of OsXIP and RXyn2 (*gray line*). *B*, SDS-PAGE analysis of each elution peak obtained in A. *C*, dose-response curve and *IC*_50_ value of RXyn2 inhibition for OsXIP_R155A. Error bars represent standard deviations of triplicate average values. *D*, hydrolysis of RBB-xylan by RXyn2 in the absence (*closed circles*) and presence of OsXIP_R155A (*open circles*) or of OsXIP (*crosses*). RBB-xylan, Remazol brilliant blue-xylan.

Since the guanidyl group of Arg155_OsXIP_ was found to form salt bridges with the carboxyl groups of the catalytic residues of RXyn2, Glu93_RXyn2_ and Glu184_RXyn2_, and a hydrogen bond with the hydroxyl group of Tyr95_RXyn2_, respectively, these interactions were speculated to critically contribute to the inhibitory activity and the binding ability to RXyn2. When an equimolar mixture of OsXIP_R155A and RXyn2 was analyzed by gel filtration chromatography, one major peak with a minor shoulder peak were obtained (peaks 1 and 2 in Fig. 7*A*). Although the major peak fraction exhibited two protein bands in SDS-PAGE, OsXIP_R155A and RXyn2 (Fig. 7*B*), the peak was eluted later than the OsXIP/RXyn2 complex (peak 5) but earlier than OsXIP_R155A and RXyn2. The shoulder one (peak 2) eluted at a position identical to that of RXyn2 and exhibited only one protein band corresponding to RXyn2 in SDS-PAGE (Fig. 7, *A* and *B*). OsXIP_R155A showed inhibitory activity against RXyn2 when *p*NP-X2 was used as substrate; however, the *IC*_50_ value was determined to be 21.2 ± 3.0 μM, which was about 60-fold larger than that of the WT (Fig. 7*C*). When RBB-xylan was used as substrate instead of *p*NP-X2, the reduction in the hydrolytic activity of RXyn2 in the presence of OsXIP_R155A was less than that in the presence of the WT (Fig. 7*D*). These results indicated that the guanidyl group of Arg155 contributes significantly to the binding ability and inhibitory activity of OsXIP on RXyn2.

## Discussion

*Rhizopus*, a genus of phytopathogenic fungi, causes rice seedling blight, resulting in extensive rice yield loss (36, 37, 38). *R. oryzae* is a soil-borne pathogen of rice and produces many carbohydrate digesting enzymes (39). Some of these enzymes are undoubtedly involved in degrading rice cell walls during the infection process. GH10 and 11 xylanases (RXyn1 and RXyn2) have been identified in culture filtrates of *R. oryzae*, we targeted these enzymes to evaluate the inhibitory activity of OsXIP (30). On the other hand, *OsXIP* was first identified as an L-ascorbic acid (AsA)-responsive gene in rice. Tokunaga and Esaka reported that several stress-related genes were induced by external AsA treatment, and *OsXIP* was found to be one of the most highly induced genes using microarray analysis (28). The expression of *OsXIP* was also drastically induced by wounding and methyl jasmonate treatment. After a decade, Sun *et al.* generated OsXIP-overexpressing transgenic rice plants showing lower disease severity upon *Magnaporthe oryzae* infection compared with WT plants (40). *M*. *oryzae* also secretes GH10 and GH11 xylanases into the medium; therefore, OsXIP appeared to be involved in self-defense in rice plants against phytopathogenic fungi by inhibiting their xylanases. These physiological findings prompted us to investigate the interactions between OsXIP and fungal xylanases.

### Inhibitory activity of OsXIP against GH10 and GH11 xylanases

Interaction analyses using gel filtration, ultracentrifugation, and ITC confirmed the formation of a complex between OsXIP and RXyn2, but not with RXyn1 (Figs. 3, 4 and S2). In gel filtration chromatography, the OsXIP/RXyn2 complex was eluted at a retention volume of 67 ml, corresponding to a globular protein of 39 kDa, which is not consistent with the theoretical molecular mass of the complex (53.6 kDa). Since the crystal structure of the OsXIP/RXyn2 complex was in a gourd-shape, the Stokes radius of the complex is unlikely to correlate with its molecular mass, probably resulting in more delayed elution than expected. We concluded that OsXIP is a unique XIP-type inhibitor that acts against only GH11 xylanases. Sequencing the rice genome revealed that it contains 18 putative GH10 and no GH11 genes. Since OsXIP did not inhibit the activity of GH10 RXyn1, it may not function as an inhibitor of endogenous rice GH10 xylanases but exclusively acts against GH11 xylanases from phytopathogens. In fact, OsXIP did not inhibit rice endogenous xylanases (41).

### Thermodynamics of OsXIP binding to GH10 and GH11 xylanases

The thermodynamic parameters obtained for binding of OsXIP to RXyn2 indicated that the binding was driven by both favorable enthalpy and entropy changes (Δ*H*° = −5.0 ± 0.20 kcal/mol and −*T*Δ*S*° = −6.5 ± 0.55 kcal/mol), resulting in a free energy change (Δ*G*°) of −11.5 ± 0.36 kcal/mol (*K*_d_ = 4.2 ± 3.3 nM), where electrostatic and hydrophobic interactions play vital roles. Dissection of the entropic term revealed that a solvation entropy change (−*T*Δ*S*_solv_°) was favorable, −26.2 ± 0.61 kcal/mol, but a conformational entropy change (–*T*Δ*S*_conf_°) was unfavorable, 17.3 ± 1.2 kcal/mol. On the other hand, Flatman *et al.* conducted similar experiments using XIP-I and a GH11 xylanase from *Aspergillus niger* at a different buffer condition (McIlvaine buffer pH 5.5) and reported that the Δ*G*° value was −10.1 kcal/mol (*K*_d_ = 41.5 ± 6.15 nM), indicating a lower binding affinity of XIP-I toward the *A. niger* GH11 enzyme than that of OsXIP toward RXyn2 by 1.4 kcal/mol (17). In addition, the binding of XIP-I was driven by a large favorable enthalpy change (Δ*H*° = −11.6 kcal/mol) with a smaller unfavorable entropic effect (−*T*Δ*S*° = 1.49 kcal/mol). Two entropic parameters for XIP-I binding to *A. niger* GH11 xylanase were −*T*Δ*S*_solv_° = −25.2 kcal/mol and –*T*Δ*S*_conf_° = 24.3 kcal/mol (17). In both cases, the flexibility of L*α*4*β*5 upon binding was reduced, which may have caused the decrease in conformational entropy. However, the interaction between OsXIP and RXyn2 resulted in significantly less loss of conformational entropy change than the interaction between XIP-I and *A. niger* GH11 enzyme. Despite of the determinations at slightly different pHs, comparison between the two datasets suggested that the binding mode of OsXIP to RXyn2 was distinct from that of XIP-I binding to *A. niger* GH11 xylanase.

### Crystal structure of OsXIP

Although the crystal structure of OsXIP adopted a (*β*/*α*)_8_-barrel fold in a similar manner to those of hevamine and XIP-I, some structural differences were found between these proteins. In particular, the loop between *α*4 and *β*5 extruded from the opposite side of the DxDxE catalytic site in hevamine (L*α*4*β*5_hev_, magenta, Fig. 5*B*) consisting of only four amino acid residues (Gly148-Val151), while the corresponding loop in OsXIP (L*α*4*β*5_OsXIP_) was made of eight amino acid residues (Asp153-Val160) (brown, Figs. 5*C* and S1). The corresponding loop of XIP-I (L*α*4*β*5_XIP-I_) is longer and consists of 11 amino acid residues (His146-Leu156) that contains Arg149 (light green, Fig. 5, *B* and *C*), whose side chain was shown to be involved in the interaction with the side chains of the two catalytic glutamic residues of XYNC (Glu85 and Glu176). Although OsXIP possessed Arg155 at the corresponding position, electron density maps of the residues making up a part of L*α*4*β*5_OsXIP_ (Arg155-Val158) were not observed, indicating L*α*4*β*5_OsXIP_ being more conformationally flexible than L*α*4*β*5_XIP-I_. Furthermore, as reported previously, XIP-I has Lys234 in helix *α*7 (light green, Fig. 5, *A* and *B*, and *D*), which forms a salt bridge with the acid-base catalyst Glu131 and water-mediated hydrogen bond with the nucleophile Glu239 of XLNC, a GH10 xylanase from *A. nidulans*, to inhibit its catalytic activity (33). On the other hand, OsXIP and hevamine were found to possess Glu237 and Asp233 at the corresponding position instead of lysine, respectively, suggesting that these proteins are unable to bind to GH10 xylanases and inhibit their activity (Fig. 5*D*). In fact, OsXIP neither bound to RXyn1 (GH10) nor inhibited its xylanase activity. In our experiment, two rice GH18 chitinases, Oschib1, and Oschib2, which have short L*α*4*β*5 as hevamine and Glu at the position corresponding to Lys234 of XIP-I, respectively, did not inhibit the activity of RXyn1 and RXyn2. These results suggest that the lengths (at least >4 amino acids) and the basic residue (arginine or lysine) corresponding to Arg149_XIP-I_ in the L*α*4*β*5 and lysine residue corresponding to Lys234_XIP-I_ in the helix *α*7 are the structural requirements for XIP to function as an inhibitory protein for both GH10 and GH11 enzymes among the proteins belonging to the GH18 family. Of note, amino acid sequence analysis of the putative rice XIPs revealed that some had a short L*α*4*β*5 and others lacked lysine residue at the position corresponding to Lys234 in the helix α7 of XIP-I. These are thought to have lost their inhibitory activity against GH11 and GH10 xylanase, respectively (Table S1).

### Crystal structure of OsXIP/RXyn2 complex

The structure of the OsXIP/RXyn2 complex revealed that the cylindrical cavity formed by the inner *β*-sheet (palm) of RXyn2 jelly roll was upright and stacked on the loops at the N terminal ends of the *β*-strands of OsXIP. On the other hand, in the complex structure of XIP-I and GH11 xylanase from *T. funiculosus* (XYNC), the cavity of XYNC laid tangentially to the part of the corresponding region of XIP-I through the loop L*α*4*β*5_XIP-I_ (Fig. 8, *A*–*D*). We designated the former as I-formation and the latter as T-formation, as schematically represented in Figure 8*F*. In an I-formation, the barrel axis of OsXIP and the central axis of the cylindrical cavity of RXyn2 lie on the same line within the OsXIP/RXyn2 complex. In a T-formation, these axes of XIP-I and XYNC intersect vertically within the XIP-I/XYNC complex.

Close inspection of L*α*4*β*5_OsXIP_ and L*α*4*β*5_XIP-I_ revealed that their structures are quite different. In OsXIP, following the C terminus of *α*-helix 4 (*α*4_OsXIP_), the 3_10_-helix 2 (*η*2_OsXIP_) consisting of four amino acid residues (Asp149-Lys152) and L*α*4*β*5_OsXIP_ consisting of eight amino acid residues (Asp153-Val160) were contiguously connected, and L*α*4*β*5_OsXIP_ was in a turn shape without any hydrogen bond (brown, Fig. 8*E*). On the other hand, in XIP-I, L*α*4*β*5_XIP-I_ consisting of 11 residues (His146-Leu156) was formed directly following the C terminus of *α*-helix 4 (*α*4_XIP-I_) without intervening 3_10_-helix (light green, Fig. 8*E*). L*α*4*β*5_XIP-I_ was in a twisted turn shape with three internal hydrogen bonds (data not shown). Furthermore, amino acid residues of RXyn2 involved in the interaction with L*α*4*β*5_OsXIP_ were completely conserved in XYNC (Fig. S4, *A* and *B*), whereas amino acid sequence identity between OsXIP and XIP-I over their entire regions falls below 40% and the 2 L*α*4*β*5 sequences share only one identical amino acid residue (Fig. S1). From these facts, we speculated that the different molecular arrangements observed in the OsXIP/RXyn2 (I-formation) and XIP-I/XYNC (T-formation) complex structures can be ascribed to structural differences between the inhibitor proteins, especially in the nontwisted L*α*4*β*5_OsXIP_ and twisted L*α*4*β*5_XIP-I_. Páez *et al.* conducted an extensive analysis of 162 GH11 sequences and discovered that the amino acid residues corresponding to Trp26, Glu93, Tyr95, Glu184, and Tyr178 in RXyn2 are strictly conserved (71%-100%) among GH11 enzymes. These residues were found to interact with OsXIP and xylotriose within the catalytic cleft. By contrast, the amino acid residues of OsXIP that interact with RXyn2 are not strictly conserved among XIPs (Fig. S1). Therefore, XIP binding to GH11 enzymes may not be restricted to the I- and T-formations. Furthermore, the binding modes of L*α*4*β*5_OsXIP_ to RXyn2 and L*α*4*β*5_XIP-I_ to XYNC were quite similar to that of xylotriose to the glycon binding site of RXyn2, suggesting that the interaction of the L*α*4*β*5s of XIP-type inhibitors with GH11 xylanases mimic the binding mode of xylotriose to subsites −3, −2, and −1 of the enzymes (Fig. S4*C*).

Mutational analysis of OsXIP revealed that the mutation of Arg155 to Ala brought about a significant loss of the inhibitory activity but the OsXIP_R155A mutant still retained the binding ability to RXyn2 (Fig. 7, *A* and *B*). From these results, although the mutation deprived the direct salt bridges and hydrogen bonds formed between the side chains of the Arg155 and the amino acid residues (Glu93, Tyr95, and Glu184) in the catalytic center of RXyn2, L*α*4*β*5_OsXIP_ of the mutant still appeared to bind and occupy subsites −3, −2, and −1 of RXyn2. In fact, OsXIP_R155A and RXyn2 were found to form a complex by gel filtration chromatography (Fig. 7*A*). The elution of OsXIP_R155A/RXyn2 complex was delayed as compared with the OsXIP/RXyn2 complex; therefore, this mutation may have created a dynamic exchange state between the monomeric and complex structures of the two proteins.

Tahir *et al.* characterized the N117A mutant of GH11 xylanase of *A. niger* and revealed that Asn117, which is located adjacent to its thumb and highly conserved in GH11 enzymes, was involved in the interaction with XIP-I (42). Although the mutant exhibited enzymatic and structural properties similar to those of the WT, XIP-I lost the ability to bind to the mutant and showed no inhibition. Notably, N^δ2^ of Asn123_XYNC_ (corresponding to Asn117 of *A. niger* GH11) formed firm hydrogen bonds with carbonyl oxygen atoms of three amino acid residues of XIP-I, Gly179_XYNC_, Phe181_XYNC_, and Ala214_XYNC_, in the XIP-I/XYNC complex structure (T-formation, Fig. S4*E*). However, Asn131 of RXyn2 (corresponding to Asn117 of *A. niger* GH11) was located outside of the interface and not involved in the interaction with OsXIP in the OsXIP/RXyn2 complex structure (I-formation, Fig. S4*D*). OsXIP may have adapted to bind and inhibit GH11 enzymes, which do not have an Asn residue corresponding to Asn117 of *A. niger* GH11 xylanase and are resistant to the inhibition by “XIP-I type” inhibitor proteins, by alternating its binding mode from T-formation to I-formation. The diversity in molecular arrangements between plant XIP-type inhibitor proteins and GH11 xylanases may provide an effective strategy for host defense against phytopathogens in various crop species.

## Experimental procedures

### Materials

*Escherichia coli* strain 10 G cells and SHuffle T7 cells were obtained from Lucigen and New England Biolabs, respectively. pRham N-His and C-His vectors were purchased from Lucigen. Ni-NTA agarose was the product from Qiagen. HiPrep 16/60 Sephacryl S-100 and HiLoad 16/600 Superdex 75 pg were obtained from GE Healthcare. *p*-Nitrophenyl *β*-D-xylobioside (*p*NP-X2), xylohexaose (X6), and beechwood xylan were obtained from Megazyme. 4-*O*-methyl-D-glucurono-D-xylan RBB-xylan was purchased from Sigma-Aldrich. Other reagents were of analytical grade and commercially available.

### Gene cloning and plasmid construction

Synthetic genes encoding the mature form of OsXIP (Ala22-Leu293), RXyn1 (Gly24-Leu331), and RXyn2 (Leu20-Ser216) were obtained from Integrated DNA Technologies (IDT). The GenBank (http://www.ncbi.nlm.nih.gov/Genbank/) accession numbers for OsXIP, RXyn1 and RXyn2 are XM_015782930, KF640266, and KF640267, respectively (28, 30). Nucleotide sequences of the genes were optimized for better expression in *E. coli* without changing the amino acid sequences. The expression vectors for these proteins fused with the (His)_6_ tag, pRham-CHis-OsXIP, pRham-NHis-RXyn1, and pRham-NHis-RXyn2, were constructed using the Expresso Rhamnose Cloning and Expression System in accordance with the manufacturer's instructions. After confirmation of the DNA sequences, pRham-CHis-OsXIP, pRham-NHis-RXyn1, and pRham-NHis-RXyn2, were introduced into *E. coli* SHuffle T7, *E. coli* 10G, and *E. coli* 10G, respectively.

### Protein expression and purification

*E. coli* cells harboring the plasmid, pRham-CHis-OsXIP, pRham-NHis-RXyn1, or pRham-NHis-RXyn2, were grown to attain 0.6 absorbance at 600 nm before induction with 0.2% (w/v) rhamnose. After induction, growth was continued for 16 h at 18 °C. The cells were harvested by centrifugation, suspended in a 10 mM Tris–HCl buffer, pH 8.0, and disrupted with a sonicator. After cell debris was removed by centrifugation at 14,000*g* for 15 min, the supernatant was dialyzed against the same buffer and applied onto a Ni-affinity column (1 × 3 cm) equilibrated with the dialysis buffer. The column was washed with the same buffer, and adsorbed proteins were eluted with a 10 mM Tris–HCl buffer, pH 8.0, containing 250 mM imidazole. The fractions eluted were pooled and further separated using a column of Sephacryl S-100 HR equilibrated with 10 mM Tris–HCl buffer, pH 8.0, containing 0.1 M NaCl. Fractions exhibiting a single protein band on SDS-PAGE were collected as purified recombinant protein (43). The protein concentration was determined by measuring absorbance at 280 nm using extinction coefficients for individual proteins (49,850 for OsXIP, 51,910 for RXyn1 and 55,350 for RXyn2, respectively) obtained from the equation proposed by Pace *et al.* (44). The mutated proteins, OsXIP_R155A and RXyn2_E184Q, were prepared and purified as with their WT proteins.

### Gel diffusion assay

A mixture of 1 g of beechwood xylan and 1 g agar was dissolved in 100 ml McIlvaine buffer, pH 6.0, and sterilized by autoclaving at 15 pounds per square inch for 20 min. After cooling (50 °C), 25 ml of the solution was poured into a sterilized Petri dish (90 mm). Filter paper discs containing 500 pmol of RXyn1, RXyn2, OsXIP, or the mixtures of these proteins (500 pmol each) in 10 μl of distilled water were placed on the xylan-agar gel at 20 mm from the center of the Petri dish. The plate was incubated for 18 h at 37 °C and then photographed. If the sample being tested shows xylanase activity, a zone of clearance (halo) appeared around the disc.

### Xylanase activity

Xylanase activity was spectrophotometrically measured using *p*NP-xylobioside (*p*NP-X2) and RBB-xylan as substrates. For the *p*NP assay, the enzyme solution (2 μl) was added to 0.2 ml of 5 mM *p*NP-X2 in 20 mM sodium acetate buffer, pH 5.0. After incubating the reaction mixture at 37 °C for 15 min, the reaction was terminated by addition of 0.8 ml of 0.25 M NaOH and absorbance of the released *p*NP at 405 nm was measured. One enzyme unit was defined as the amount of enzyme required to release 1 nmol of *p*NP per min at 37 °C. The Michaelis constant (*K*_m_) was determined by plotting the initial velocity as a function of *p*NP-X2 concentration. The experimental data were then fitted to the Michaelis–Menten equation using GraphPad Prism version 10.0 (GraphPad Software). For the experiment with RBB-xylan, the enzyme solution (20 μl) was added to 0.2 ml of 0.2% (w/v) RBB-xylan in 20 mM sodium acetate buffer, pH 5.0. After incubating the reaction mixture at 37 °C for 15 min, the reaction was terminated by addition of 0.78 ml of 95% (v/v) ethanol. The reaction mixture was spun down and the absorbance of the resulting supernatant containing the soluble digested xylan fraction at 590 nm was measured.

### Inhibition studies

Inhibition of RXyn2 was assayed by measuring the enzymatic activity using *p*NP-X2 as a substrate in the presence of OsXIP. OsXIP was added to reaction mixture containing 1 mM *p*NP-X2 and 0.6 μM RXyn2 at concentrations ranging from 0 to 1.0 μM. The inhibitory concentration leading to 50% activity loss (*IC*_50_) was obtained by fitting experimental data to the logistic curve using KaleidaGraph (Synergy Software). To determine the mechanism of inhibition, Dixon plots were created using *p*NP-X2 with three different concentrations ranging from 0.5 to 1.0 mM. The concentrations of the inhibitor in the assay were 0.1, 0.2, 0.3, and 0.4 μM. The reciprocal initial velocities were plotted against the inhibitor concentration for each substrate concentration, and the data were analyzed using the Dixon method (45). The inhibition constant (*K*_i_) value was calculated using the Cheng-Prusoff equation for competitive inhibition, with the *IC*_50_ and *K*_m_ values (46, 47, 48).

### Isothermal titration calorimetry experiments

Isothermal titration calorimetry (ITC) experiments were performed using an iTC_200_ system (MicroCal) at 25 °C. Protein and buffer solutions were thoroughly degassed before their use to avoid air bubbles in the calorimeter and improve results. The OsXIP solution in 20 mM sodium acetate buffer, pH 5.0, was loaded into a syringe, whereas the RXyn1 or RXyn2 solution in the identical buffer, pH 5.0, was placed in the reaction cell of volume 0.2028 ml. For all titrations, 2 μl aliquots were injected into the sample cell with a stirring speed of 1000 rpm. The titrations were completed after 20 injections. The integration of heat pulses and model-fitting were performed with Origin 7.0 using a single-site binding model. Individual datasets obtained from the titration experiments fitted well to the theoretical curves, providing the stoichiometries (*n*), equilibrium association constants (*K*_a_) and enthalpy changes (Δ*H*°) of the protein–ligand interactions. The binding free energy change (Δ*G*°) and entropy change (Δ*S*°) were calculated from the relationship as follows,

$$\Delta G\circ =-RT\;\cdot \;\ln\;K_{a}=\Delta H\circ -T\Delta S\circ$$

For examining the temperature dependence, ITC measurements were performed at varied temperatures from 20 to 40 °C. The Δ*H*° values obtained for various temperatures were plotted against temperatures, and the slope of a straight line fitted to the experimental points corresponds to the heat capacity change (Δ*C*_p_°). As the entropy of solvation is regarded as zero for proteins near 385 K, Δ*C*_p_° was converted to the solvation entropy change (Δ*S*_solv_°) at 25 °C (298 K) according to the following relationship,

$$\Delta S_{\text{solv}}{}^{\circ}=\Delta C_{p}{}^{\circ}\;\ln\left(\frac{298.15K}{385.15K}\right)$$

The conformational entropy change (Δ*S*_conf_°) was calculated from Δ*S*°, the solvation entropy change (Δ*S*_solv_°) obtained in this study and the mixing entropy change (Δ*S*_mix_°, − 8 cal K^−1^ mol^−1^), based on the following equation (49),

$$\Delta S{}^{\circ}=\Delta S{{}^{\circ}}_{\text{solv}}+\Delta S{{}^{\circ}}_{\text{mix}}+\Delta S{{}^{\circ}}_{\text{conf}}$$

To examine the interaction between X6 and RXyn2_E184Q, a 5.0 mM solution of X6 was titrated into a RXyn2_E184Q solution (18.5 μM).

### Gel filtration column chromatography

To investigate the complex formation between RXyn1 or RXyn2 and OsXIP, analytical gel filtration was performed with an ÄKTA FPLC system (GE Healthcare) system using a HiLoad 16/600 Superdex 75 pg at 4 °C. The column was first equilibrated with 20 mM sodium acetate buffer, pH 5.5, containing 0.3 M NaCl following injection of the samples (1.5 ml). The flow rate was set to 0.5 ml/min. Elution profiles were monitored by UV absorption at 280 nm and calibrated to a low molecular weight standard. The final concentration of each protein was 9 μM. Equimolar concentrations of RXyn1 or RXyn2 and OsXIP (4.5 μM each) were mixed and incubated for 30 min at 4 °C prior to the analysis.

### Analytical ultracentrifugation

Sedimentation velocity experiments were carried out at 20 °C in a ProteomeLab XL-I analytical ultracentrifuge (Beckman Coulter) equipped with double-UV and Rayleigh interference detection. The purified proteins (9 μM each) were centrifuged at 42,000 rpm using an AN60-Ti rotor and 12 mm thick epon double sector centerpieces. Absorbance and interference profiles were recorded every 5 min. Buffer viscosity (η = 1.021 cP) and density (ρ = 1.0050° g/ml) at 20 °C were estimated with SEDNTERP 1.09 (50). Partial specific volumes at 20 °C were estimated based on amino acid sequences using SEDNTERP 1.09 software. Data were analyzed with SEDFIT 15.3 using a continuous size distribution *c*(*s*) model and Svedberg software (51, 52). The sedimentation coefficient was measured in the Svedberg (S) unit where 1 S = 10^−13^ sec.

### Crystallization and data collection

Crystallization conditions for OsXIP, RXyn2, and OsXIP/RXyn2 complex were screened using the sparse-matrix sampling method using sitting drop vapor diffusion at 293 K. Drops were prepared by mixing 1 μl protein solution with 1 μl precipitant solution and were equilibrated against 100 μl reservoir solution. The protein concentration used for crystallization of OsXIP and RXyn2 was 5.0 mg/ml. For crystallization of the OsXIP/RXyn2 complex, a mixture of these proteins (5.0 mg/ml each) was prepared. Crystals of OsXIP and OsXIP/RXyn2 grew within 3 weeks, but no crystals of RXyn2 were generated. OsXIP was crystallized under 0.1 M Bis-Tris pH 5.5 and 2.0 M ammonium sulfate. For X-ray data collection, crystals were dipped into a cryoprotectant solution consisting of the reservoir solution containing 20% (v/v) glycerol. Crystals of OsXIP/RXyn2 complex grew under 0.1 M Bis-Tris pH 5.5 and 25% PEG 3350 in the presence of synthetic zeolite molecular sieves, a hetero-epitaxic nucleant. The crystals were soaked briefly in cryoprotectant solution consisting of the reservoir solution containing 20% (v/v) glycerol. After cryocooling the crystals at 95 K, X-ray diffraction data were collected at a wavelength of 0.97865 Å on the beamline BL-17A at the Photon Factory using an EIGER X 16 M (Dectris). The data were integrated and scaled with XDS (53). The processing statistics are summarized in Table 1.

### Structural determination and refinement

The structures of OsXIP and OsXIP/RXyn2 were solved by the molecular replacement method using the program PHASER (54), where the structures of XIP-I and XYNC in the XIP-I/XYNC complex structure (PDB ID, 1TE1) served as search models, respectively (33). The models were improved by several rounds of refinement with PHENIX (55) and COOT programs (56). The structure of OsXIP was refined to an *R*_work_/*R*_free_ of 16.6/17.9% at a resolution of 1.3 Å. The final model contains one protein molecule that includes 272 residues and 244 water molecules. The stereochemistry of the model was verified using MolProbity (57), showing 98.1%, 1.9%, and 0% of protein residues in the favored, allowed, and disallowed regions of the Ramachandran plot, respectively. For the OsXIP/RXyn2 complex, two protein complex molecules were in the crystallographic asymmetric unit. The structure of OsXIP/RXyn2 was refined to an *R*_work_/*R*_free_ of 26.7/31.7% at a resolution of 2.2 Å. The final model contained two protein molecules that include 272 residues for OsXIP, 197 residues for RXyn2 and 276 water molecules. The stereochemistry verification showed 95.0%, 5.0%, and 0% of protein residues in the corresponding regions, respectively. Molecular graphics were illustrated with PyMol software (http://www.pymol.org/). The refinement statistics are summarized in Table 1. The atomic coordinates and structural factors have been deposited in the PDB, under accession codes 9K1M (OsXIP) and 9K1N (OsXIP/RXyn2 complex).

## Data availability

The data generated in this study are available upon reasonable request from the corresponding author.

## Supporting information

This article contains supporting information.

## Conflict of interest

The authors declare that they have no conflicts of interest with the contents of this article.

## Supporting information

Supporting Information_Table S1

9K1M.pdb

9K1N.pdb
