# Supplementary material for: Molecular arrangements that accompany binding of rice xylanase inhibitor protein OsXIP and the Rhizopus oryzae GH11 xylanase RXyn2
Source: J Biol Chem. 2025 Jun 16;301(8):110385. doi: 10.1016/j.jbc.2025.110385 (PMC12329526; doi:10.1016/j.jbc.2025.110385)
Supplement: Supporting Information (Figure) [file mmc1.docx]

**Supporting Information**

**Molecular arrangement in the complex structure of the rice xylanase inhibitor protein OsXIP and the *Rhizopus oryzae* GH11 xylanase RXyn2.**

Takayuki Ohnuma^1,2^, Jun Tanaka^1^, Harutada Ozaki^1^, Keigo Mitsui^1^, Daichi Tsujitsugu^1^, Miki Okugawa^1^, Toru Takeda^1^, Makoto Ihara^3^, Tamo Fukamizo^1,4^, Daijiro Takeshita^5^

^1^Department of Advanced Bioscience, Kindai University, 3327-204 Nakamachi, Nara 631-8505, Japan

^2^Agricultural Technology and Innovation Research Institute, 3327-204 Nakamachi, Nara 631-8505, Japan

^3^Department of Applied Biological Chemistry, Kindai University, 3327-204 Nakamachi, Nara 631-8505, Japan

^4^School of Biomolecular Science and Engineering (BSE), Vidyasirimedhi Institute of Science and Technology (VISTEC), Wang Chan, Rayong, 21210 Thailand

^5^Biomedical Research Institute, National Institute of Advanced Industrial Science and Technology (AIST), 1-1-1 Higashi, Tsukuba, Ibaraki 305-8566, Japan.

**Figure S1. Amino acid sequence alignment of xylanase inhibitor proteins.**

The alignment was obtained using the ClustalW program and manual correction. Identical amino acids are boxed for all sequences. Conservative amino acid substitutions are colored cyan. Dashes indicate gaps. The amino acid residues of OsXIP and XIP-I, whose side chains are directly involved in interacting with RXyn2 and XYNC, respectively, are highlighted with a black background. The secondary structures of OsXIP and XIP-I are presented at the top and bottom of the sequences and numbered from the N-terminus, respectively: *α*, *α*-Helices (black rectangles), *η*, 3_10_-helices (open rectangles), *β*, *β*-strands (gray rectangles). Open circles indicate cysteine residues, which form disulfide bonds. Closed circles indicate basic residues conserved in L*α*4*β*5 and *α*-helix 7 of XIP-I type inhibitor proteins. OsXIP (GenBank accession number AK060742); OsHI-XIP (AK062114); riceXIP (AK064356); RIXI (AK063939); XIP-I (AJ422119).


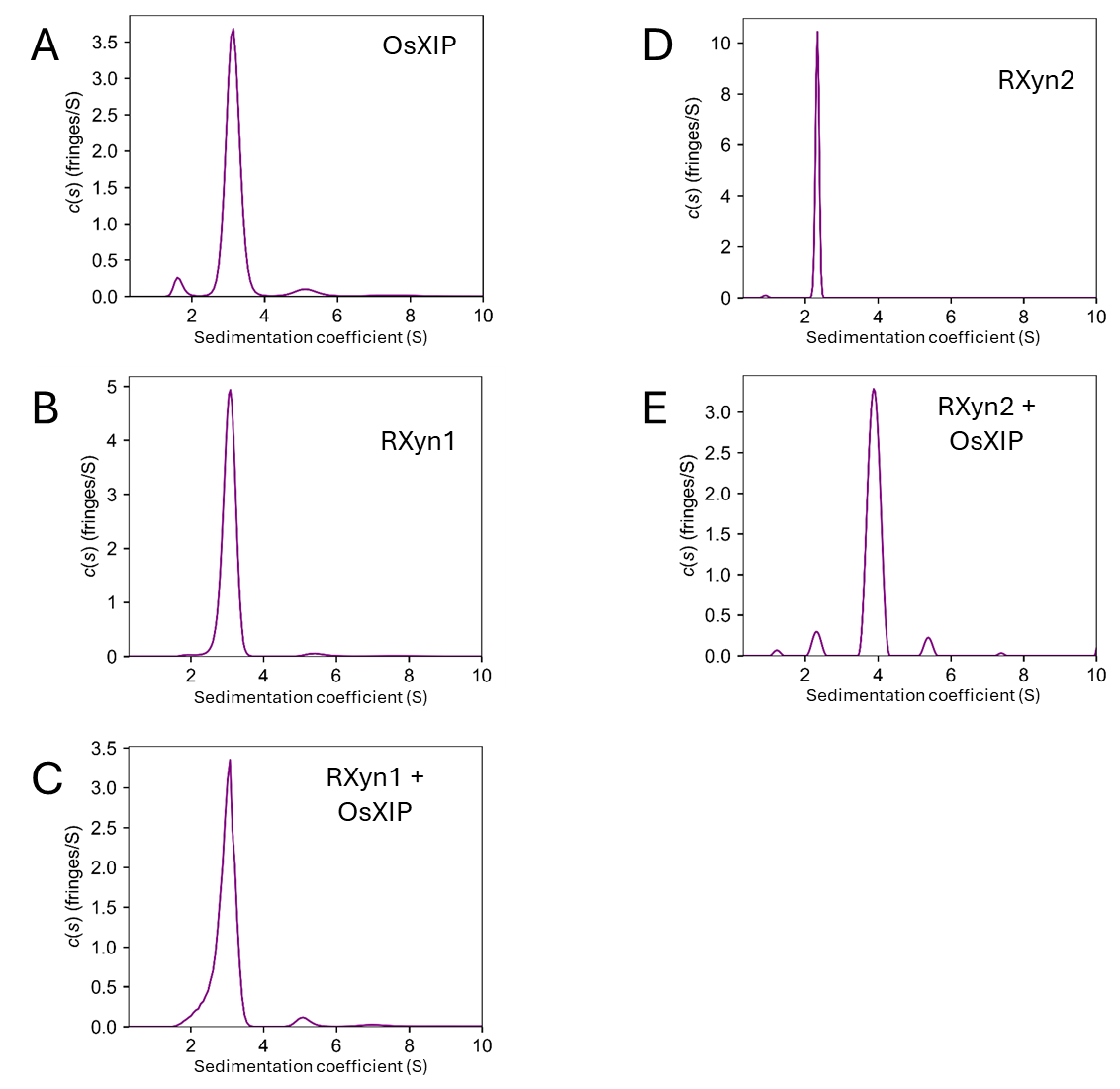


**Figure S2. Sedimentation velocity analytical ultracentrifugation analysis of OsXIP complexation.**

(A) OsXIP; (B) RXyn1; (C) An equimolar mixture of RXyn1 and OsXIP; (D) RXyn2; (E) An equimolar mixture of RXyn2 and OsXIP.


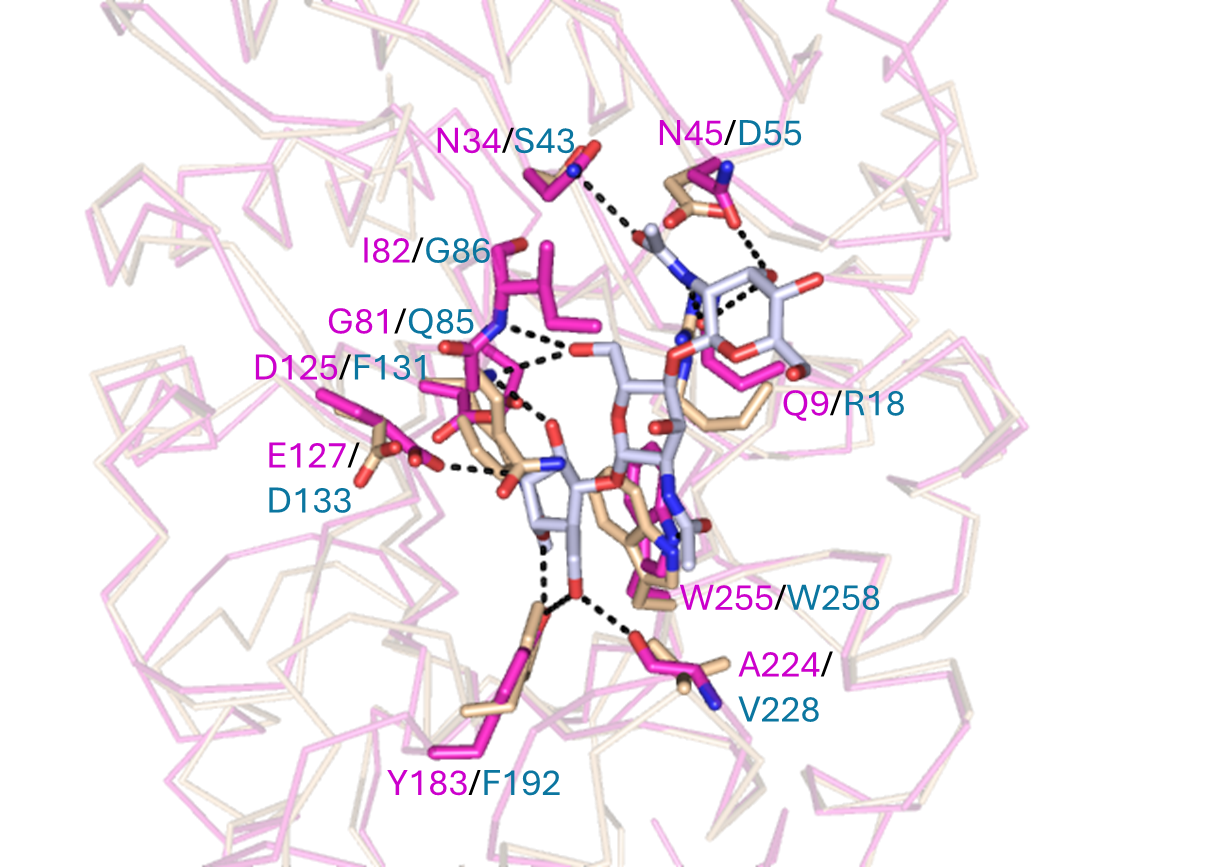


**Figure S3. Crystal structures of OsXIP and allosamidin-liganded hevamine.**

Superimposed structure of OsXIP (brown line) with allosamidin-liganded hevamine (PDB ID 1llo, magenta line). Amino acid residues of hevamine involving the interaction with allosamidin are shown using magenta sticks and labeled. Allosamidin is depicted using gray sticks. Hydrogen bonds formed between these residues and allosamidin are shown as black dashed lines. Amino acid residues of OsXIP corresponding to these residues are shown using brown sticks and labeled.

**
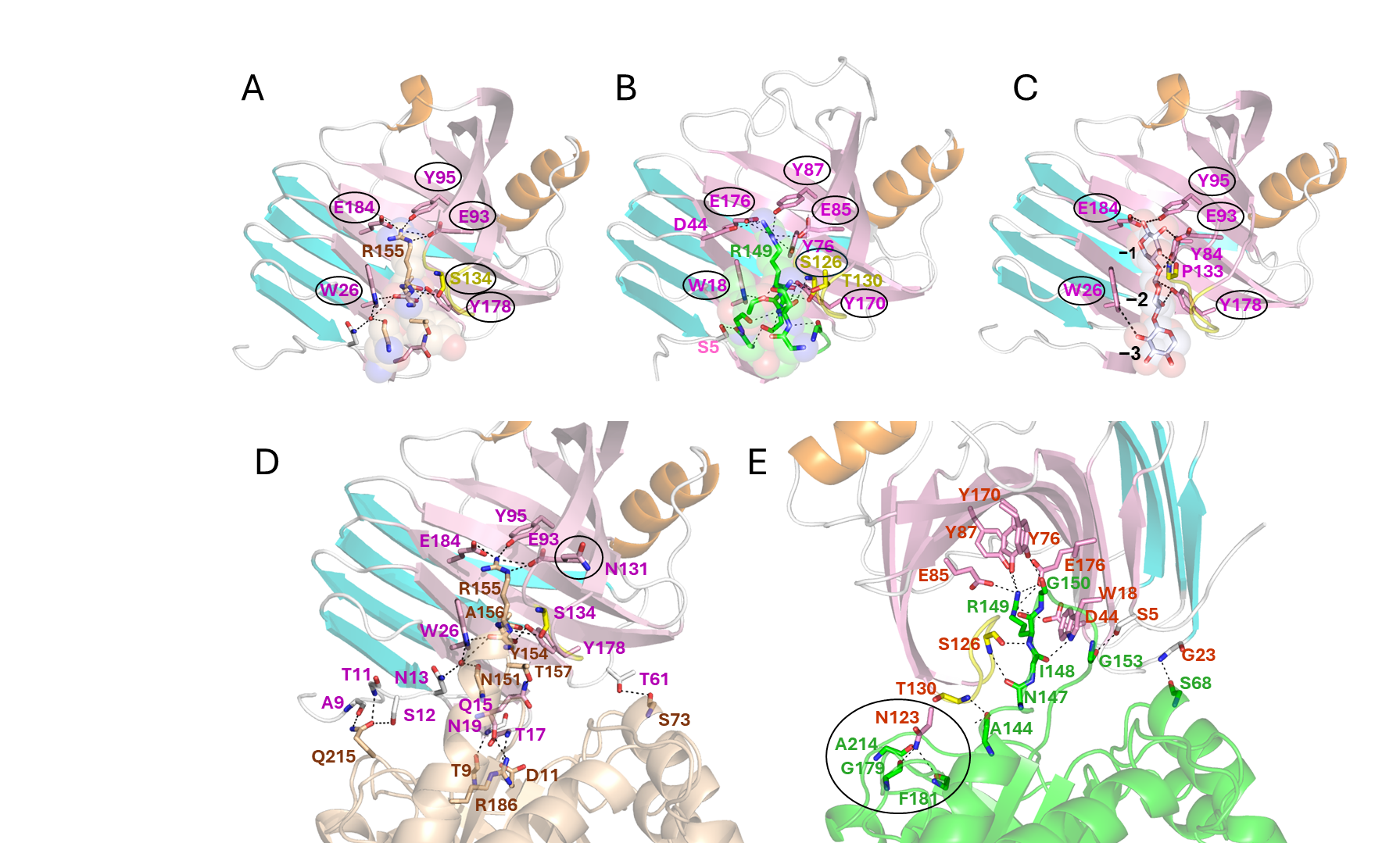
**

**Figure S4. Binding mode of L*α*4*β*5 and xylotriose to the glycon binding site of GH11 enzyme.**

(A) Detailed view of the interaction between L*α*4*β*5_OsXIP_ and the glycon binding site of RXyn2. (B) Detailed view of the interaction between L*α*4*β*5_XIP-I_ and the glycon binding site of XYNC. (C) Detailed view of the interaction between xylotriose and the glycon binding site of RXyn2. Xylotriose was superimposed from the structure of XynII liganded with xylotriose (PDB ID 6JWB). Dashed lines indicate the intermolecular hydrogen bonds in A and B. In C, dashed lines indicate the putative intermolecular hydrogen bonds. Amino acid residues involved in the interactions are shown using sticks and labeled. Conserved amino acid residues between RXyn2 and XYNC, which are involved in the interactions, are marked using circles. Amino acid residues of L*α*4*β*5_OsXIP_, L*α*4*β*5_XIP-I_, and xylotriose are also depicted in transparent spheres. (D) and (E), Localization of Asn residues (Asn131_RXyn2_ and Asn123_XIP-I_) corresponding to Asn117 of the *A. niger* GH11 xylanase in the OsXIP/RXyn2 and XIP-I/XYNC complex. Ellipses indicate the positions of the Asn residues.
