# Supplementary material for: Molecular arrangements that accompany binding of rice xylanase inhibitor protein OsXIP and the Rhizopus oryzae GH11 xylanase RXyn2
Source: J Biol Chem. 2025 Jun 16;301(8):110385. doi: 10.1016/j.jbc.2025.110385 (PMC12329526; doi:10.1016/j.jbc.2025.110385)
Supplement: Supporting Information_Table S1 [file mmc2.docx]

| Table S1. Amino acid sequences of the Lα4β5 and α7 regions of putative rice XIPs and their inhibition specificities. | | | | |  |  |
| --- | --- | --- | --- | --- | --- | --- |
|  |  |  |  | Inhibition  specificity | |  |
| Gene_ID |  | L*α*4*β*5 (GH11) | *α*7 (GH10) |  |  | Protein |
| Os11g47560 |  | HYEQLARQLHGRG--------VLLTATVRCAYPD | EQDEG-YMFQ**K**DLY | GH10 |  |  |
| Os11g47570 |  | HYDELARQLHGRG--------VALTATVRCSYPD | EQDEVGYMFQ**K**DLY | GH10 |  |  |
| Os11g47520 |  | HYDDLARRLDGYNKYY**R**GRVGVLLTATTRCSYPD | EQDSA-WMFQ**K**DLY | GH10 | GH11 | riceXIP |
| Os08g40680 |  | HYDELARRLHGYG------AGVIWTATTRCSYPD | EQDEA-WVFQ**K**DLY | GH10 |  |  |
| Os11g47550 |  | HYDELARKLYSYRN--N**K**GKGVMLTATPRCRFPD | EQDPG-YLSP**K**PLY | GH10 | GH11 |  |
| Os05g15920 |  | NYNKLAKLLYAHNKDY**R**GTVGVMLTATTRCEYPD | EVDREAYISPEDLK |  | GH11 |  |
| Os05g15880 |  | HYNELAKMLYDHNKDY**R**ATVGVMVTATTRCGYPD | DVDKDAYMPPEALN |  | GH11 | OsXIP |
| Os05g15770 |  | HYDELARRLYAHNKDY**K**GRLGVMLTATARCVFPD | EADRDGYVSH**K**DLY | GH10 | GH11 | OsHI-XIP |
| Os05g15850 |  | HYDELARLLHGHSNG-----GVMLTATARCVFPD | EADRDAYMSH**K**DLY | GH10 |  |  |
| Os11g47530 |  | HYDELARRLFSHYKF-----EMLLTATTRCSYPD | EQDANAYLPR**K**VLF | GH10 |  |  |
| Os11g47580 |  | HYDDLARNLYAYNKMY**R**ARTPVRLTATVRCAFPD | VPGKNDNVFI**K**QLY | GH10 | GH11 | RIXI |
| Os08g40740 |  | ------VKLYAYNKLY**R**ARTPVRLTATVRCMFPD | LPGKNDNVFV**K**QLY | GH10 | GH11 |  |
| Os11g47510 |  | HYDDLANRINDYNQNIHDPIGIMLTATVRCSYPD | VTGKNDMVGVGELS |  |  |  |
| Os11g47500 |  | HYDDLANRINDYNQNI**R**DPIGIMLTATVRCSYPD | LTGKNDMVAVGELR |  | GH11 |  |
| Os08g40690 |  | HYYEIAERINYDTRHW**R**DPIGFKLTATVSCAYDD | LTGKNDMVAVGELY |  | GH11 |  |
| Os11g47590 |  | NYDELAKRLGEHGG------VLLTATVRCMDGQE | DAASDGWINPAALV |  |  |  |
| Os11g47610 |  | HYDELARRLRSFGRE**K**PA-VRLTASPACSLALFD | EMS--GFVDPQTLR | | GH11 |  |
| Os11g47600 |  | NYDVLVRRLAGYRG**K**-----PVLLTATPRCVYPD | TAAD--WINPESLY | | GH11 |  |
| Os06g25010 |  | RYDVLALELAKHNI**R**GAPGKPLHLTATPRCTFPP | -D------------ | | GH11 |  |
| Os07g43820 |  | RYDVLATELAKR--G**K**PPRRALHLTATTRCAFP— | QDGRSGYVYP**K**TLY | GH10 | GH11 |  |
| XIP-I |  | RYDVLALELAKHNI**R**GGPGKPLHLTATVRCGYPP | -DKSHQWVHP**K**NVY | GH10 | GH11 | XIP-I |
| The basic amino acid residues required for inhibition of GH10 and GH11 xylanases are bolded and underlined, respectively. | | | | | |  |
